# Single-cell transcriptome sequencing reveals spatial distribution of IL34^+^ cancer-associated fibroblasts in hepatocellular carcinoma tumor microenvironment

**DOI:** 10.1038/s41698-023-00483-9

**Published:** 2023-12-11

**Authors:** Ganggang Wang, Zhijie Zhou, Wenzhi Jin, Xin Zhang, Hao Zhang, Xiaoliang Wang

**Affiliations:** Department of Hepatobiliary Surgery, Pudong Hospital, Fudan University, Shanghai, 200000 China

**Keywords:** Hepatocellular carcinoma, Hepatocellular carcinoma

## Abstract

We utilized scRNA-seq, a well-established technology, to uncover the gene expression characteristics of IL34^+^ CAFs within HCC. We analyzed the related mechanisms through in vitro and in vivo assays. To begin, we acquired scRNA-seq datasets about HCC, which enabled us to identify distinct cell subpopulations within HCC tissues. We conducted a differential analysis to pinpoint DEGs associated with normal fibroblasts (NFs) and CAFs. Subsequently, we isolated NFs and CAFs, followed by the sorting of IL34^+^ CAFs. These IL34^+^ CAFs were then co-cultured with T cells and HCC cells to investigate their potential role in Tregs infiltration, CD8^+^ T cell toxicity, and the biological processes of HCC cells. We validated our findings in vivo using a well-established mouse model. Our analysis of HCC tissues revealed the presence of seven primary cell subpopulations, with the most significant disparities observed within fibroblast subpopulations. Notably, high IL34 expression was linked to increased expression of receptor proteins and enhanced proliferative activity within CAFs, with specific expression in CAFs. Furthermore, we identified a substantial positive correlation between IL34 expression and the abundance of Tregs. Both in vitro and in vivo experiments demonstrated that IL34^+^ CAFs promoted Tregs infiltration while suppressing CD8^+^ T cell toxicity. Consequently, this promoted the growth and metastasis of HCC. In summary, our study affirms that IL34^+^ CAFs play a pivotal role in augmenting the proliferative activity of CAFs, facilitating Tregs infiltration, and inhibiting CD8^+^ T cell toxicity, ultimately fostering the growth and metastasis of HCC.

## Introduction

In the past few decades, there has been a growing recognition of the significance of the tumor microenvironment in malignant phenotypes. The traditional cancer view, centered around tumor cells, has been revised to highlight the interactions between cancer cells and their surrounding environment. This revelation has sparked the development of new concepts in targeted therapy, aimed at disrupting the paracrine signaling between different cell types within the tumor mass^1^.

Tumor-associated fibroblasts (CAFs) are the most common cellular component in the tumor microenvironment, present in various types of cancers such as breast cancer, pancreatic cancer, and hepatocellular carcinoma^2^. CAFs originate from resident fibroblasts or stellate cells within the tissue, undergoing transformation under the influence of growth factors. They contribute to the shaping of the tumor microenvironment, forming a barrier that restricts the infiltration of therapeutic immune cells, thereby reducing the effectiveness of tumor treatment. Additionally, CAFs can secrete various cytokines or metabolic products that impair immune cell function and promote tumor development, invasion, and metastasis. CAFs exhibit heterogeneity and can be classified into multiple subtypes based on their functional differences. While cellular morphology is the most reliable method for distinguishing CAFs, commonly used cell markers such as alpha-smooth muscle actin (α-SMA) and fibroblast-specific protein-1 (FSP-1) are neither exclusive nor entirely indicative, thus increasing the possibility that CAFs are composed of various subpopulations of cells^2^.

Interleukin-34 (IL34) is a newly discovered cytokine that interacts with three receptors: colony-stimulating factor 1 receptor (CSF1-R), CD138, and PTP-ζ. It plays a crucial role in regulating the differentiation and function of various target cells. IL-34 is primarily produced by macrophages, endothelial cells, fibroblasts, and neuronal cells^3^. Existing evidence suggests that IL-34 is involved in viral infections, autoimmune diseases, and cancer development. High IL-34 expression is associated with poor survival rates and tumor recurrence in patients with HCC. However, the specific mechanisms by which IL-34 influences the occurrence and metastasis of HCC remain unclear^4^.

Single-cell sequencing is an innovative technology that has unveiled the hidden cellular subsets within large amounts of RNA sequencing data. This technique is commonly employed to determine heterogeneity in cell populations and molecular profiles^5^. Consequently, we utilized single-cell sequencing to uncover the gene spatial distribution and expression characteristics of IL34^+^ CAFs, aiming to investigate their molecular mechanisms in promoting HCC growth and metastasis.

## Results

### HCC tissue contains seven main cell subpopulations

HCC is the second leading cause of cancer-related deaths worldwide. It has a high incidence rate and poor prognosis, accounting for 75–85% of all cases^6^. Over the past few decades, the importance of the tumor microenvironment in malignant phenotypes has been gradually revealed. CAFs are the most common cellular component of the tumor microenvironment and play an indispensable role in tumorigenesis and tumor progression^7^.

To analyze HCC-related scRNA-seq data, we obtained datasets from the Gene Expression Omnibus (GEO), including three adjacent normal tissue samples (GSM5709316, GSM5709324, GSM5709329) from GSE189903, two HCC samples (GSM5076749, GSM5076750) from GSE166635, and one HCC sample (GSM6435354) from GSE210679. We used the Seurat package to integrate the data. Firstly, we examined the number of genes (nFeature_RNA), mRNA molecules (nCount_RNA), and the percentage of mitochondrial genes (percent.mt) in all cells of the scRNA-seq data. The results showed that the majority of cells had nFeature_RNA < 5000, nCount_RNA < 20000, and percent.mt <20% (Supplementary Fig. 1A). After filtering out low-quality cells with 200 <nFeature_RNA < 5000 and percent.mt <20%, we obtained an expression matrix with 24,371 genes and 71,662 cells. The correlation analysis of sequencing depth indicated that the filtered data had a correlation coefficient of *r* = 0.12 between nCount_RNA and percent.mt, and *r* = 0.91 between nCount_RNA and nFeature_RNA (Supplementary Fig. 1B), suggesting that the filtered cell data had good quality for further analysis.

**Fig. 1:**
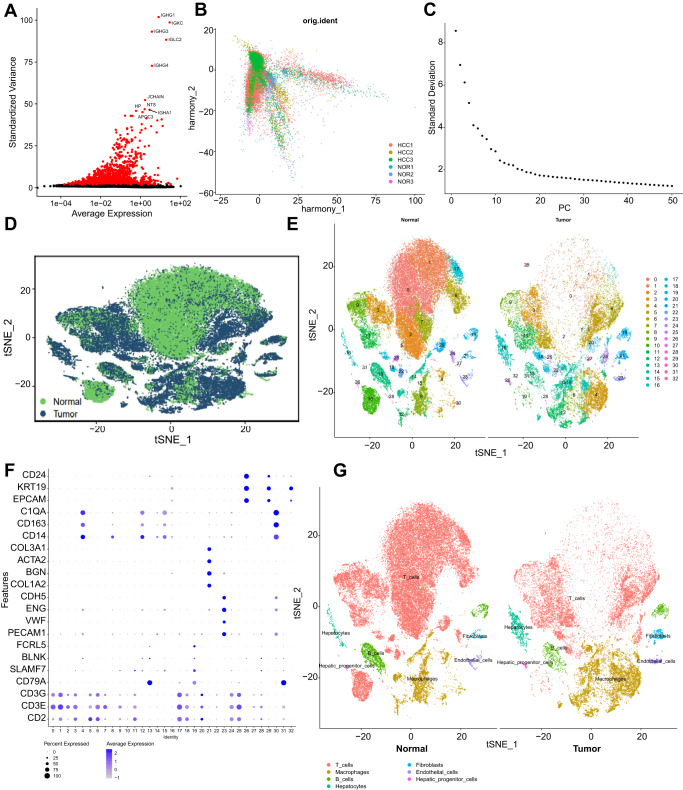
Cell clustering and annotation of scRNA-seq data. Note: **A** Differential expression analysis to identify highly variable genes. Red indicates the top 2000 highly variable genes, while black represents genes with low variability. The top 10 genes in the highly variable gene set are labeled. **B** Cell distribution in PC_1 and PC_2 after batch correction using Harmony. Each point represents a single cell. **C** Distribution of standard deviations for principal components (PCs). PCs with larger standard deviations are more important. **D** tSNE visualization of cell clustering, showing the aggregation and distribution of cells from both normal adjacent samples and HCC samples in two dimensions. Green represents normal adjacent samples, while deep blue represents HCC samples. **E** tSNE visualization of cell clustering, displaying the aggregation and distribution of cells from different sources. Each color represents a cluster. **F** Expression of known lineage-specific marker genes in different clusters. Darker blue indicates higher average expression levels, and larger circles represent more cells expressing the gene. **G** tSNE visualization of cell annotation based on clustering, with each color representing a cell subpopulation.

Subsequently, we analyzed the filtered cells, selected highly variable genes based on gene expression variance, and chose the top 2000 genes for downstream analysis (Fig. 1A). We calculated the cell cycle using the CellCycleScoring function (Supplementary Fig. 1C) and normalized the data. Then, we performed linear dimensionality reduction using principal component analysis (PCA) on the selected highly variable genes. Here, we presented the heat map of the main associated gene expressions in PC_1 - PC_6 (Supplementary Fig. 1D) and the cell distribution in PC_1 and PC_2 (Supplementary Fig. 1E), revealing evident batch effects among the samples.

To eliminate the batch effects and improve the accuracy of cell clustering, we used the harmony package for batch correction of the samples (Supplementary Fig. 1F). The results showed that the sample batch effects were effectively eliminated (Fig. 1B). Meanwhile, we used ElbowPlot to rank PCs by standard deviation (Fig. 1C), indicating that PC_1 - PC_20 adequately reflected the information contained in the selected highly variable genes and had good analytical significance.

Furthermore, we performed nonlinear dimensionality reduction using the t-distributed stochastic neighbor embedding (tSNE) algorithm on the top 20 PCs. Through clustering, we identified 32 clusters and obtained the marker gene expression profiles for each cluster (Fig. 1D, E). We annotated the cells using known cell lineage-specific marker genes obtained from relevant literature and the online database CellMarker (Fig. 1F)^8^. We identified a total of seven cell types: B_cells, T_cells, endothelial cells, fibroblasts, macrophages, hepatic progenitor cells, and hepatocytes (Fig. 1G). Specifically, clusters 13, 19, and 31 were B_cells, clusters 0, 1, 2, 3, 5, 6, 7, 9, 10, 11, 17, 18, 20, 24, 22, 25, 27, 28 were T_cells, cluster 23 was endothelial cells, cluster 21 was fibroblasts, clusters 4, 8, 12, 14, 15, and 30 were macrophages, clusters 26 and 32 were hepatic progenitor cells, and cluster 16 was hepatocytes. Compared to the adjacent normal samples, HCC tissues exhibited a significant decrease in T_cells and an increase in fibroblasts and macrophages.

In summary, our results revealed that the HCC samples and their adjacent normal samples could be divided into 32 clusters, comprising 7 cell subtypes. Notably, HCC tissues showed a marked decrease in T_cells and an increase in fibroblasts and macrophages.

### Significant differences in the molecular characterization of Fibroblasts in standard tissue samples adjacent to cancer and HCC samples

CAFs are the most common cells in the tumor microenvironment, present in breast cancer, pancreatic cancer, and HCC^2^. CAFs are transformed from intrinsic fibroblasts or stromal cells in the tissue under the stimulation of growth factors. They shape the extratumoral mechanisms, form barriers to drug penetration and therapeutic immune cell infiltration, and reduce the effectiveness of tumor treatment. CAFs can also secrete various cytokines or metabolic products to inhibit immune cell function and promote tumor development, invasion, and metastasis^5^. Therefore, CAFs are selected as the primary research target for subsequent studies.

**Fig. 2:**
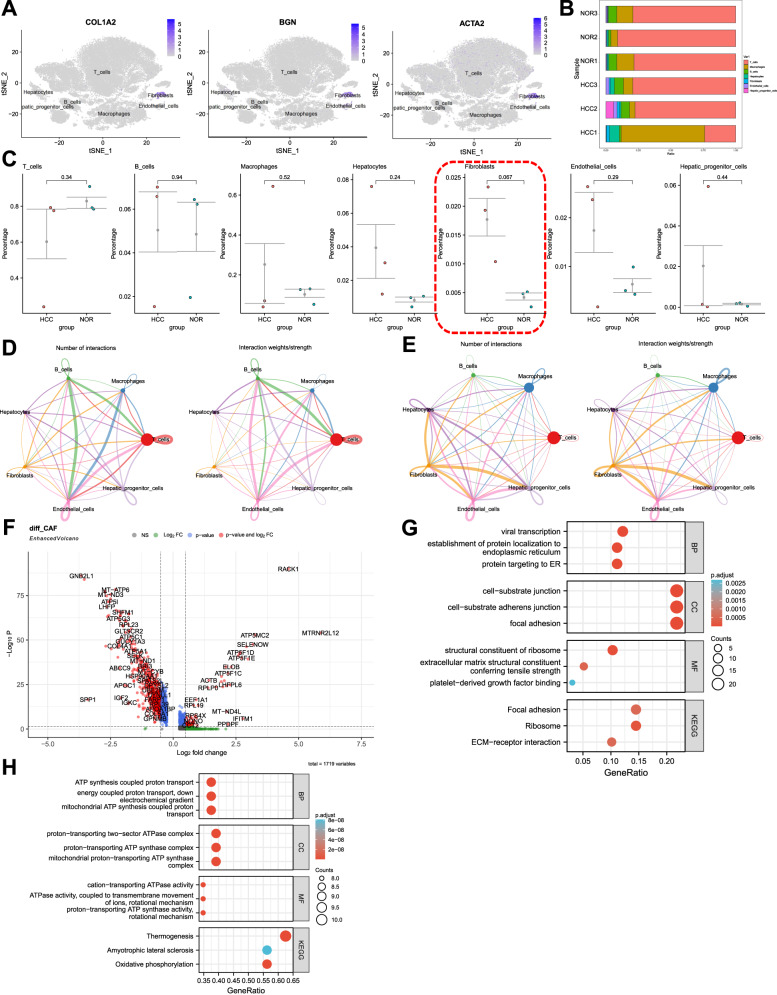
Molecular characterization of fibroblasts in adjacent normal samples and HCC samples. Note: **A** Expression patterns of fibroblast markers in different cell subgroups, where darker blue indicates higher average expression levels. **B** Proportions of different cell subgroups in each sample, represented by different colors. **C** P-values obtained from T-test analysis assessing differences in cell abundance between adjacent and HCC samples. HCC represents tumor samples, and NOR represents adjacent normal samples. **D** Communication network of cells in adjacent normal samples, where the thickness of the lines on the left denotes the number of pathways and the thickness of the lines on the right represents the interaction strength. **E** Communication network of cells in HCC samples, where the thickness of the lines on the left denotes the number of pathways and the thickness of the lines on the right represents the interaction strength. **F** Volcano plot showing differential gene expression between fibroblasts in adjacent normal and HCC samples. Red dots to the left of the dashed line represent genes with high expression in HCC samples, while dots to the right represent genes with low expression in HCC samples. **G** Bubble plot depicting GO and KEGG enrichment for highly expressed genes in HCC samples. The size of the circles represents the number of genes enriched in that pathway, and the color represents the relevance of enrichment for that pathway. **H** Bubble plot depicting GO and KEGG enrichment for lowly expressed genes in HCC samples. The size of the circles represents the number of genes enriched in that pathway, and the color represents the relevance of enrichment for that pathway.

To investigate the variations of fibroblasts in the tumor adjacent and HCC samples, we first validated the annotation of fibroblasts (Fig. 2A). Subsequently, using the Seurat package, we calculated the proportions of different cell types in individual samples (Fig. 2B), and conducted T-test analysis to determine the differential cell proportions between tumor adjacent and HCC samples. The results showed that fibroblasts had the lowest *P*-value in terms of cell proportion differences between tumor adjacent normal samples and HCC samples, indicating a more significant difference compared to other cell types (Fig. 2C).

Furthermore, to explore the functional differences of fibroblasts in tumor adjacent and HCC samples, we utilized the R package “CellChat” to investigate the pathway activities between different cell types. The results revealed that compared to tumor-adjacent normal tissues, fibroblasts in HCC tissues exhibited stronger interactions with macrophages, B cells, T cells, and hepatic progenitor cells, indicating enhanced intercellular communication (Fig. 2D, E).

Moreover, we performed differential gene expression analysis on fibroblasts in tumor-adjacent normal samples and HCC samples, identifying 162 significantly upregulated genes and 26 significantly downregulated genes in HCC samples (Fig. 2F). These genes were subjected to gene enrichment analysis. The GO enrichment results showed that the upregulated genes in CAFs from HCC samples were mainly enriched in biological processes such as viral transcription, establishment of protein localization to the endoplasmic reticulum, and protein targeting to the endoplasmic reticulum. On the other hand, the downregulated genes in CAFs from HCC samples were mainly enriched in biological processes such as ATP synthesis coupled proton transport, energy coupled proton transport down the electrochemical gradient, and mitochondrial ATP synthesis coupled proton transport (Fig. 2G, H). The KEGG enrichment results indicated that the upregulated genes in CAFs from HCC samples were mainly enriched in signaling pathways such as focal adhesion and ribosome, while the downregulated genes were mainly enriched in signaling pathways such as thermogenesis and amyotrophic lateral sclerosis (Fig. 2G, H).

These results indicate that, compared to tumor-adjacent normal samples, the abundance of CAFs in HCC samples is significantly increased, and their interaction with T cells, B cells, and other cell types is notably enhanced.

### High IL34 expression promotes the proliferation and activity of CAFs

According to literature, it has been reported that IL34 binds to receptors such as CSF1-R and PTP-ζ, regulating the differentiation and functionality of fibroblasts^3^. To investigate whether IL34 regulates the tumor microenvironment mediated by CAFs, CAFs and adjacent NFs were isolated from HCC tissues of patients who underwent surgical resection. Firstly, the quality of obtained NFs and CAFs was assessed by immunofluorescence staining for the expression of endothelial cell marker CD31, epithelial cell marker Cytokeratin, and fibroblast marker Vimentin. The results indicated that CD31 and Cytokeratin were almost not expressed in the obtained fibroblasts, while Vimentin showed significant expression (Supplementary Fig. 2A, B). This suggests that the obtained fibroblasts had minimal contamination from epithelial cells and endothelial cells, and were of good quality for subsequent analysis.

**Fig. 3:**
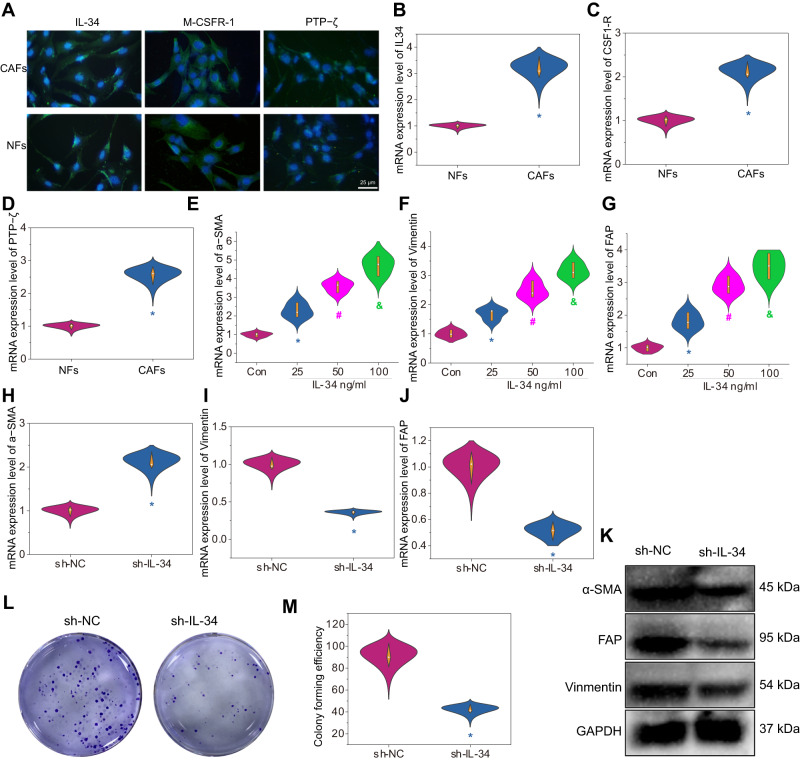
The effect of IL34 expression levels on the activity and proliferation capacity of CAFs. Note: **A** Immunofluorescence detection of IL34, CSF1-R, and PTP-ζ receptor expression in CAFs and NFs. IL34, CSF1-R, and PTP-ζ are shown in green, and DAPI is shown in blue. Scale bar represents 75 µm. **B** Quantification of IL34 expression levels in tumor fibroblasts using RT-qPCR. **C** Quantification of CSF1-R expression levels in tumor fibroblasts using RT-qPCR. **D** Quantification of PTP-ζ expression levels in tumor fibroblasts using RT-qPCR. **E** Quantification of α-SMA expression levels in tumor fibroblasts treated with different concentrations of IL34 using RT-qPCR. **F** Quantification of Vimentin expression levels in tumor fibroblasts treated with different concentrations of IL34 using RT-qPCR. **G** Quantification of FAP expression levels in tumor fibroblasts treated with different concentrations of IL34 using RT-qPCR. **H**–**J** RT-qPCR analyses of α-SMA, FAP, and Vimentin expression levels in CAFs after IL34 silencing. **K** Western Blot analysis of α-SMA, FAP, and Vimentin protein expression levels in CAFs after IL34 silencing. **L**, **M** Plate colony assays to evaluate the proliferation capacity of CAFs after IL34 silencing. * represents a significant difference compared to the control group, NFs, or sh-NC group at *P* < 0.05. # represents a significant difference compared to the 25 ng/ml group at *P* < 0.05. & represents a significant difference compared to the 50 ng/ml group at *P* < 0.05. All cell experiments were repeated three times.

Subsequently, RT-qPCR and immunofluorescence were used to investigate the expression of IL34, CSF1-R, and PTP-ζ in the obtained CAFs and NFs. The results showed that IL34 was significantly expressed in CAFs compared to NFs, and the expression of its functional receptors CSF1-R and PTP-ζ also increased significantly (Fig. 3A–D), indicating that IL34 and its functional receptors were significantly higher in CAFs than in NFs.

It has been reported that IL34 can induce the activation phenotype of fibroblasts in colorectal cancer^3^. To study whether the high expression of IL34 affects the activation phenotype of HCC fibroblasts, CAFs were stimulated with three concentrations of IL34 protein (25, 50, and 100 ng/ml), with untreated CAFs as the control (addition of an equal concentration of BSA solution). The RT-qPCR results showed a significant increase in the RNA levels of α-SMA, Vimentin, and fibroblast activation protein (FAP) with increasing IL34 concentration (Fig. 3E–G). Furthermore, the knockdown of IL34 expression in CAFs was validated by lentiviral transduction (Supplementary Figs. 2C, D, 9, 10). Through RT-qPCR and western blot experiments, the expression of activation phenotype proteins in CAFs after knockdown and the changes in their proliferative capacity were investigated. The results showed that IL34 knockout significantly decreased the expression of α-SMA, Vimentin, and FAP (Fig. 3H–K, Supplementary Figs. 5–8), as well as significantly reduced the proliferative capacity of CAFs (Fig. 3L, M).

### Gene expression of IL34^+^ CAFs in HCC samples is closely associated with lymphocytes

According to the literature, IL34 is primarily produced by macrophages, endothelial cells, fibroblasts, and neurons. High expression of IL34 is associated with lower survival rates and tumor recurrence in HCC patients, although the specific mechanisms underlying its impact on HCC occurrence and metastasis remain unclear^3^.

**Fig. 4:**
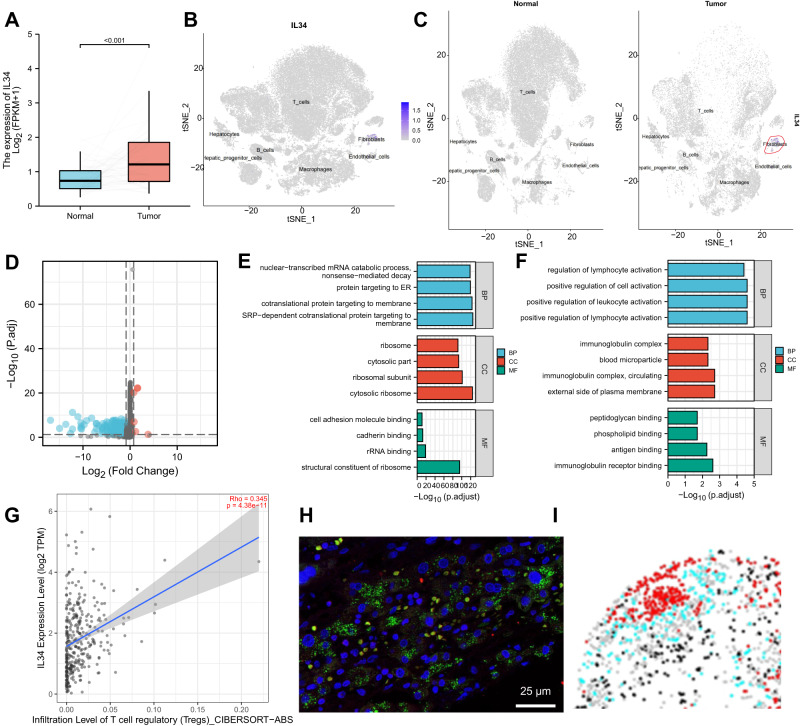
The expression pattern and characterization of IL34 in CAFs. Note: **A** Box plot of IL34 expression levels in adjacent normal samples and HCC samples from TCGA transcriptome data, including 50 adjacent normal tissue samples and 374 HCC tissue samples; **B** Expression pattern of IL34 in different cell subtypes, with deeper blue indicating higher expression levels; **C** Expression pattern of IL34 in NFs derived from adjacent normal samples and CAFs derived from HCC samples, with deeper blue indicating higher expression levels; **D** Volcano plot showing differentially expressed genes between IL34+ CAFs and IL34^-^ secretory CAFs, with blue representing genes significantly downregulated in IL34+ CAFs and red representing genes significantly upregulated in IL34+ CAFs; **E** Bar chart showing GO enrichment analysis results for downregulated genes in IL34+ CAFs, with blue, red, and green representing BP, CC, and MF, respectively; **F** Bar chart showing GO enrichment analysis results for upregulated genes in IL34+ CAFs, with blue, red, and green representing BP, CC, and MF, respectively; **G** Correlation between IL34 expression levels and Tregs content; **H** Immunofluorescence staining of CAFs and Tregs marker proteins in HCC tissue, with yellow indicating Foxp3, green indicating COL1A2, and blue indicating DAPI; **I** Corresponding phenotypic chart of CAFs and Tregs, with red representing Tregs and cyan representing CAFs.

To further investigate the influence of IL34-mediated CAFs on HCC, we examined the expression levels of IL34 using HCC RNA-seq data from the TCGA database. We found significantly higher expression of IL34 in HCC patients (Fig. 4A). Additionally, through analysis of single-cell sequencing data, we observed high expression of IL34 in the fibroblast cell population (Fig. 4B), whereas its expression was low in normal fibroblasts (Fig. 4C), indicating specific expression of IL34 in HCC CAFs.

To explore the characteristics of genes associated with high IL34 expression, we extracted a gene expression matrix of CAFs from HCC samples in the Seurat analysis. This matrix included 455 CAFs, which were divided into IL34 high-expression CAFs (IL34^+^) and IL34 low-expression CAFs (IL34^-^) based on the median IL34 expression level. Differential gene expression analysis was performed, and we selected genes with a P-adjust <0.05 and a logFC >0.85. We identified 5 significantly up-regulated genes and 194 significantly down-regulated genes (Fig. 4D). Gene ontology (GO) enrichment analysis revealed that the down-regulated genes in IL34^+^ CAFs were mainly associated with biological processes such as cotranslational protein targeting to the membrane and SRP-dependent cotranslational protein targeting to the membrane (Fig. 4E), while the up-regulated genes were primarily enriched in biological processes related to regulation of lymphocyte activation, positive regulation of cell activation, positive regulation of lymphocyte activation, and positive regulation of leukocyte activation (Fig. 4F). These results suggest a correlation between IL34 high-expression genes in CAFs and lymphocyte metabolic activity. Furthermore, using the online tool TIMER 2.0, we investigated immune infiltration and found a significant positive correlation between IL34 expression and Treg cell infiltration (Fig. 4G).

To further explore the relationship between IL34^+^ CAFs and Tregs, we collected HCC tissue from patients and performed immunofluorescence staining for the fibroblast marker protein COL1A2 and the Treg marker protein Foxp3. We found that CAFs had a higher density around Tregs (Fig. 4H, I), suggesting a close relationship between CAFs and Treg regulation.

In summary, our results indicate that IL34 is highly expressed in CAFs in HCC samples, and the high-expression gene profiles of IL34^+^ CAFs are closely associated with Treg immune infiltration processes.

### IL34^+^ CAFs promote the differentiation of Tregs and thereby inhibit CD8^+^ T cell toxicity

According to the literature, CAFs can promote tumor progression by mediating immune infiltration. In our study, bioinformatics analysis revealed that highly expressed genes in CAFs are correlated with lymphocyte immune processes, and IL34 is positively associated with Tregs cell infiltration.

**Fig. 5:**
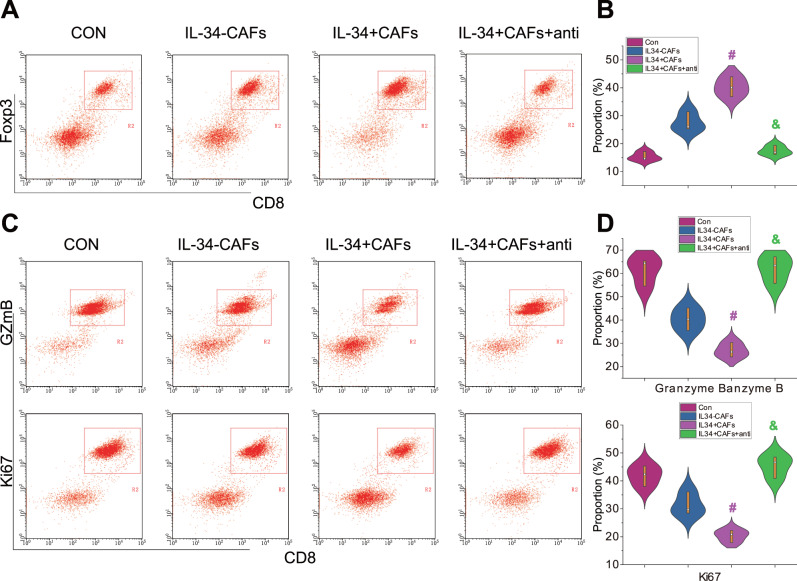
Influence of IL34^+^ CAFs on the Cytotoxicity of CD8^+^ T Cells. Note: **A**, **B** Representative results and quantification of Foxp3 expression in T cells detected by flow cytometry; **C**, **D** Representative results and quantification of GzmB and Ki67 expression, cytotoxicity markers, in CD8^+^ T cells detected by flow cytometry. # denotes statistical significance compared to the IL34-CAFs group (*P* 0.05), & denotes statistical significance compared to the IL34+ CAFs group (*P* < 0.05); all cellular experiments were repeated three times.

To investigate the impact of CAFs on the differentiation of Tregs cells, we performed flow cytometry to select IL34^+^ CAFs and IL34^-^ CAFs, and then co-cultured them with T cells. The expression of the Tregs cell marker protein Foxp3 was detected using flow cytometry to determine the proportion of Tregs cells (Supplementary Fig. 3). Compared to the control group, the IL34^-^ CAFs group showed a slight increase in Tregs cells, but not significantly. On the other hand, the IL34^+^ CAFs group exhibited a significant increase in Tregs cells compared to the IL34^-^ CAFs group. Additionally, the addition of IL34-anti led to a significant decrease in the proportion of Tregs cells (Fig. 5A, B).

Tregs cells primarily mediate cytotoxic T lymphocyte immune processes. To further investigate, we co-cultured the Tregs cells treated with CAFs with CD8^+^ T cells and analyzed the expression of the cytotoxic markers Granzyme B (GzmB) and Ki67 using flow cytometry^9^. The results showed that the IL34^-^ CAFs group exhibited a slight, but not significant, decrease in the expression of GzmB and Ki67 compared to the control group. In contrast, the IL34^+^ CAFs group showed a significant decrease in the expression of GzmB and Ki67 compared to the IL34^-^ CAFs group. Furthermore, co-culturing with IL34-anti resulted in a significant increase in the expression of GzmB and Ki67 compared to the IL34^+^ CAFs group (Fig. 5C, D).

### IL34^+^ CAFs promote the in vivo tumorigenic and metastatic capacity of HCC cells

**Fig. 6:**
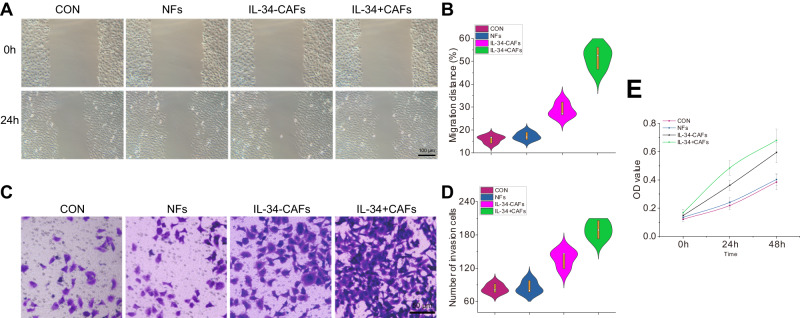
Impact of IL34 + CAFs on the proliferation, migration, and invasion abilities of HCC cells. Note: **A**, **B** Scratch assays were performed to evaluate the migration ability of HCC cells among different treatment groups. **C**, **D** Transwell assays were conducted to assess the invasion ability of HCC cells in different treatment groups (scale bar=50 μm). **E** CCK-8 assays were used to measure the proliferation ability of HCC cells. * represents *P* < 0.05 compared to the NFs group, # represents *P* < 0.05 compared to the IL34-CAFs group. Each cellular experiment was repeated three times.

To investigate the impact of IL34^+^ CAFs on the growth and metastasis of HCC cells, we co-cultured IL34^+^ CAFs, IL34- CAFs, or NFs with human HCC cells. After allowing the cells to stabilize, we assessed the differences in proliferation, migration, and invasion ability of HCC cells using CCK-8, scratch assay, and Transwell methods. The results demonstrated that HCC cells co-cultured with IL34^+^ CAFs exhibited significantly higher proliferation, migration, and invasion abilities compared to the other groups. There were no significant differences observed between the NFs co-culture group and the untreated control group. Furthermore, there was no significant difference between the IL34- CAFs group and the NFs group (Fig. 6A–E). These findings indicate that IL34^+^ CAFs have the potential to significantly enhance the proliferation, migration, and invasion abilities of HCC cells.

### IL34 affects CD8^+^ T-cell toxicity by promoting Tregs infiltration to influence HCC tumor growth and metastasis

**Fig. 7:**
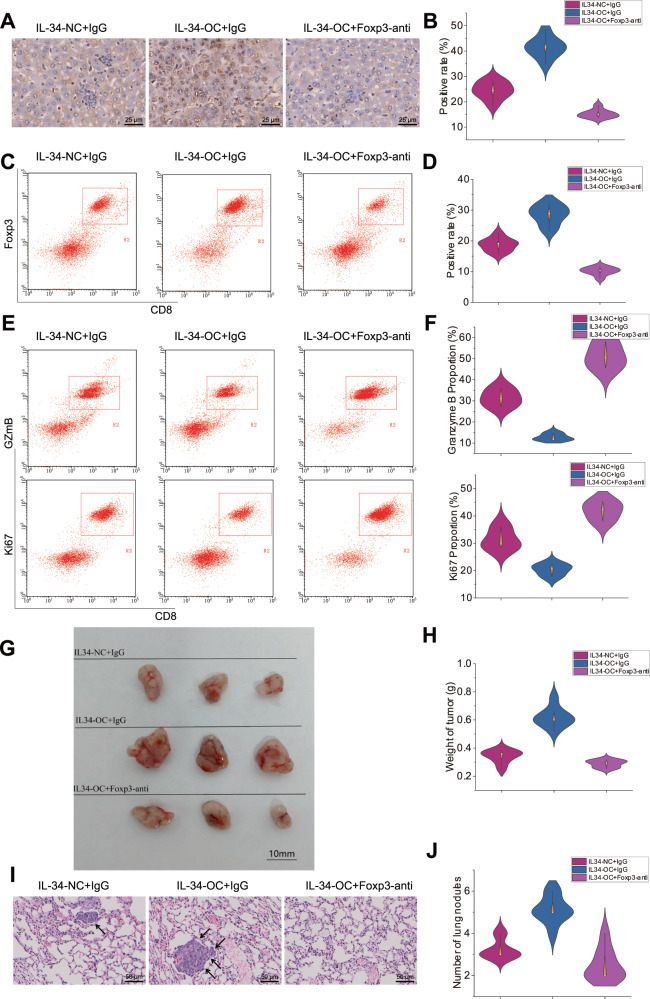
In vitro experiments to validate the impact of IL34 + CAFs on tumor progression. Note: **A** Immunohistochemistry was employed to examine the expression of Treg markers in HCC tissues, with a scale of 25 µm; **B** Statistical graph depicting the positive rate of immunohistochemistry; **C**, **D** Flow cytometry was conducted to detect the content of Tregs in HCC tissues; **E**, **F** Flow cytometry was utilized to evaluate the expression of the cytotoxic protein Granzyme B (GzmB) and Ki67 in CD8^+^ T cells of HCC tissues; **G** Comparison of tumor volume in mice from different treatment groups; **H** Statistical comparison of tumor weight in mice from different treatment groups; **I**, **J** H&E staining was conducted to examine lung metastasis in mice from each group (×100), with black arrows indicating areas of pathological tissue. * represents a significant difference compared to the IL34-NC+IgG group at a *p*-value of less than 0.05, # represents a significant difference compared to the IL34-OC+IgG group at a *p*-value of less than 0.05. Each group of mice experiments consisted of 8 mice.

To further validate the involvement of IL34^+^CAFs in promoting Tregs infiltration and impacting tumor growth and metastasis of CD8^+^ T cells, we utilized lentiviral-mediated overexpression of IL34 in CAFs (Supplementary Figs. 4, 11, 12) and mixed them with mouse tumor cells to establish a mouse model of HCC. After two weeks, we collected HCC tissues and performed immunohistochemical staining to detect the expression of Tregs markers. Additionally, flow cytometry analysis was used to determine the proportion of Tregs. The results showed a significant increase in Foxp3 expression and Tregs proportion in HCC tissues with IL34 overexpression compared to the control group. Furthermore, the addition of Foxp3 antibody resulted in a significant reduction in Foxp3 expression and Tregs proportion (Fig. 7A–D), indicating that IL34 can promote Tregs infiltration in HCC tissues.

Subsequently, we employed flow cytometry analysis to examine the expression of cytotoxic markers, Granzyme B (GzmB) and Ki67, in CD8^+^ T cells^9^. The results revealed a significant decrease in the expression of cytotoxic markers in CD8^+^ T cells with IL34 overexpression compared to the control group. However, when IL34 overexpression was combined with inhibition of Tregs function, the expression of cytotoxic markers in CD8^+^ T cells was significantly higher than in the IL34 overexpression group (Fig. 7E, F). These findings suggest that IL34^+^ fibroblasts suppress the cytotoxicity of CD8^+^ T lymphocytes by promoting Tregs.

Furthermore, to investigate the impact of IL34 expression on HCC tumorigenesis, we collected tumor and lung tissues from the mouse model and recorded the volume and weight of the tumors. Hematoxylin and eosin staining was utilized to observe the effect of IL34 on the metastatic ability of HCC cells in mice. The results showed that the IL34-OC+IgG group had significantly larger tumor volumes and a greater number of lung metastatic foci compared to the IL34-NC+IgG group. However, in the IL34-OC+Foxp3-anti group, the tumor volume was significantly smaller than in the IL34-OC+IgG group, and the number of lung metastatic foci was significantly lower (Fig. 7G–J).

## Discussion

In our study, we performed bioinformatics analysis on single-cell sequencing data of both adjacent normal tissues and HCC tissues. The results revealed the presence of seven distinct cell subgroups in both the adjacent normal tissue and HCC tissue. Among these, the fibroblast subgroup showed the most significant differences in abundance between the adjacent normal tissue and HCC tissue. Therefore, we propose that fibroblasts may play a significant role in the process of HCC compared to other cell types. These findings are consistent with previous reports^8^. Furthermore, compared to adjacent normal tissues, the subpopulation of CAFs showed stronger connections with macrophages, B cells, T cells, and hepatic progenitor cells, indicating enhanced interactions. This reveals differences in the abundance and expression characteristics of tumor microenvironment cell subsets between HCC tissues and adjacent normal tissues, along with dynamic changes in cell communication. Previous literature reports have shown that CAFs chemotactically attract and regulate T cells by secreting IL6 and CXCL12, similar to what has been observed in other solid tumors^10,11^.

Further experiments demonstrated that high expression of IL34 enhances the expression of receptor proteins and proliferation activity in CAFs. Moreover, IL34 exhibits specific high expression in CAFs, as confirmed by single-cell data. By conducting differential gene analysis and immune infiltration analysis on CAFs with high and low IL34 expression, we found that the upregulated genes in IL34 high-expressing CAFs are closely associated with lymphocyte metabolism and Tregs infiltration. In vitro experiments showed that IL34^+^ CAFs enhance the proliferation, migration, and invasion capabilities of HCC cells. IL-34 interacts with three receptors: CSF1-R, CD138, and PTP-ζ, to regulate the differentiation and function of various target cells^3^. Elevated serum levels of IL-34 have been identified as an independent prognostic factor for non-viral HCC patients. High serum IL-34 levels in non-viral HCC patients have been associated with poor prognosis^12^. In other studies focusing on HBV-related HCC, IL-34 has been shown to enhance HCC proliferation and development in vivo, with noticeable increases in tumor volume and weight in IL-34-treated mice. Additionally, IL-34 has been found to promote proliferation and migration of HCC cells in vitro^13^. Liu et al. previously reported a correlation between elevated intrahepatic and serum IL-34 levels in HCC patients compared to non-liver cancer cohorts^14^. Zhou et al. discovered that the miR-28-5p-IL-34-macrophage feedback loop regulates liver cell metastasis, thereby serving as a novel prognostic factor and potential therapeutic target for hepatocellular carcinoma. Eleonora Franzè et al. reported that IL34 induced normal fibroblasts (NF) to acquire a cellular phenotype similar to that of CAF and that knockdown of IL34 in CAF reduced its tumorigenic properties and proliferation, a result that is consistent with our study^3^. In summary, it has been established that the production of IL34 can facilitate the development of HCC. Elevated levels of IL34 have been demonstrated to promote CAFs. However, there is a lack of research elucidating the spatial distribution of IL34^+^ CAFs in the tumor microenvironment of HCC, as well as their gene expression characteristics. Furthermore, the molecular mechanisms through which IL34^+^ CAFs promote HCC growth and metastasis remain to be clarified.

We further verified through in vitro experiments that the expression of IL34 promotes Tregs infiltration and inhibits CD8^+^ T cell cytotoxicity. Using an in vivo experiment with an orthotopic HCC mouse model, we found that IL34 high-expressing CAFs affect CD8^+^ T cell cytotoxicity by promoting Tregs infiltration, thereby promoting HCC cell growth and metastasis. It has been reported in the literature that CAFs secrete various cytokines or metabolites to inhibit immune cell function and promote tumor development, invasion, and metastasis^2^. In colorectal cancer, IL34 has been shown to regulate the modulators Netrin-1 and b-FGF in colorectal CAFs, thereby influencing the behavior of colorectal cancer cells. High expression of IL34 correlates closely with T cells and macrophage metabolism^3^.

**Fig. 8:**
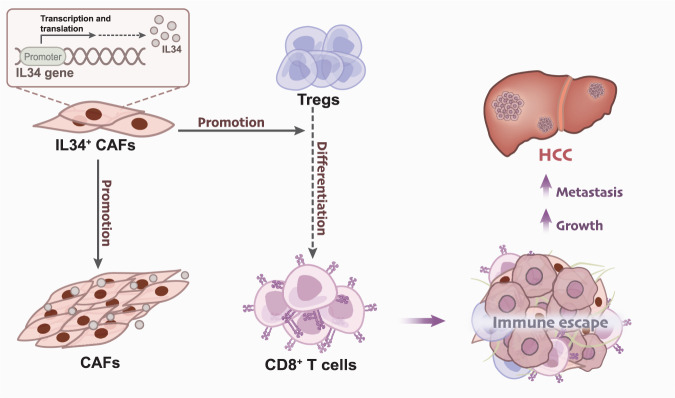
The molecular mechanism by which IL34 + CAFs promote the growth and metastasis of HCC.

Based on the above experiments, we have reached the preliminary conclusions that adjacent normal tissues and HCC tissues mainly consist of seven cell subpopulations, with IL34 being one of the genes specifically highly expressed in CAFs. IL34^+^ CAFs enhance the proliferation activity of CAFs, promote differentiation of Tregs, and suppress CD8^+^ T cell cytotoxicity, leading to immune escape and promoting the growth and metastasis of HCC (Fig. 8). Our study reveals the gene expression characteristics of IL34^+^ CAFs and the molecular mechanisms through which they promote HCC metastasis, providing important insights for HCC immunotherapy.

## Methods

### Single-cell transcriptome data acquisition

The Gene Expression Omnibus (GEO, https://www.ncbi.nlm.nih.gov/geo/) was used to access scRNA-seq data related to HCC. This included the datasets GSE189903, which consisted of three samples of normal adjacent tissues: GSM5709316 (NOR1), GSM5709324 (NOR2), and GSM5709329 (NOR3); and GSE166635, which consisted of two samples of HCC tissues: GSM5076749 (HCC1) and GSM5076750 (HCC2). Additionally, the dataset GSE210679 provided one sample of HCC tissue: GSM6435354 (HCC3). R software and the “Seurat” package were utilized to analyze the data^15^, with one HCC tissue sample GSM6435354 (HCC3) from data set GSE210679. Quality control was performed on the data using the criteria of 200 <nFeature_RNA < 5000 and percent.mt <20, and highly variable genes were selected based on the top 2000 genes with the highest variance.

Transcriptome FPKM (fragments per kilobase of transcript per million fragments mapped) data of liver HCC patients were downloaded from The Cancer Genome Atlas (TCGA) database (https://portal.gdc.cancer.gov/). This dataset included 50 samples of normal adjacent tissues and 374 samples of HCC tissues.

The Harmony software (version 0.1.0) was applied to assess and remove batch effects within each subgroup. Subsequently, the cells within each group were re-analyzed according to the standard protocol of Seurat^16,17^.

### TSNE cluster analysis

To reduce the dimensionality of the scRNA-Seq dataset, principal component analysis (PCA) was performed on the highly variable genes based on the top 2000 genes with the highest variance. The Elbowplot feature of the Seurat software package was used to select the top 20 principal components for downstream analysis. The FindClusters function provided by Seurat was employed to identify major cell subpopulations, using the default resolution (res = 1). Next, a non-linear dimensionality reduction of the scRNA-seq sequencing data was performed using the t-SNE algorithm. The Seurat software package was used to identify marker genes for various cell subpopulations. Cell annotation for the cells was conducted by combining known lineage-specific marker genes with the online resource CellMarker (http://xteam.xbio.top/CellMarker/)^8^.

### Screening of DEGs

The “limma” package in the R software was used to identify DEGs^18^. DEGs between HCC samples and adjacent normal samples in CAFs were selected based on the criteria |logFC|> 1 and *P*.adjust <0.05. DEGs between IL34^-^ CAFs and IL34^+^ CAFs were selected based on the criteria |logFC|> 0.85 and *P*.adjust < 0.05. The R software package “pheatmap” was used to generate heatmaps and volcano plots to visualize the expression of DEGs.

### GO and KEGG enrichment analysis

In this study, the DEGs obtained were subjected to GO and KEGG enrichment analysis using the “clusterProfiler” package, the “org.Hs.eg.db” package, the “enrichplot” package, and the “ggplot2” package in the R language. Subsequently, bubble plots were constructed to visualize the enrichment results of three categories in Gene Ontology (GO), namely biological process (BP), cellular component (CC), and molecular function (MF). Additionally, a bubble plot depicting the results of KEGG enrichment analysis was also generated^19^.

### Fibroblast isolation

Our study utilized tumor samples and non-tumor tissues obtained from 9 patients who underwent HCC treatment at our hospital between January 2019 and January 2021. Before surgery, all patients did not receive radiotherapy or chemotherapy. All subjects signed written informed consent before participating in our study. The study has been approved by the Ethics Committee of Shanghai Pudong Hospital (QWJWLX-01) and complies with the Declaration of Helsinki.

Collected tumor and non-tumor tissues were washed twice with pre-cooled PBS solution containing 2% gentamicin (15140148, Thermofisher, USA) to remove blood clots and necrotic tissue from the tissue surface. After cutting the tissues into small pieces with scissors, they were added to a solution of 0.1% type IV collagenase (17104019, Thermofisher, USA) containing 10% fetal bovine serum (16140089, Thermofisher, USA). This mixture was then transferred to a centrifuge tube and digested at a constant temperature of 37 °C for 30–40 minutes on a shaker. The digestion medium, along with any remaining tissues, was passed through a 200-mesh sieve, and the filtrate was centrifuged at 4 °C for 5 minutes (50 × *g*) and the supernatant was discarded. The pellet was resuspended in a complete DMEM medium (11965092, Thermofisher, USA) and washed twice. Red blood cells were removed using red blood cell lysis buffer (C3702-120 ml; Beyotime, China), and the cell density was adjusted to 10^6^/ml. Based on the differences in fibroblast growth rate and adhesion ability compared to other cells, the cell suspension was added to the first well of a 6-well plate and allowed to incubate for 20 minutes. The cells adhering to the plate were predominantly fibroblasts, and the supernatant was aspirated and transferred to the second well for a further 20 minutes of incubation. This process was repeated, and the cells adhering to the two wells were agitated with DMEM containing 10% fetal bovine serum, and then cultured and passaged in a humidified incubator at 37 °C and 5% CO_2_, with media changes every 3 days. Immunofluorescence detection was performed to identify fibroblast marker protein expression in the obtained fibroblasts. Mouse CAFs were obtained by isolating tumor tissues from an in situ mouse HCC model (two weeks).

### Cell treatment

The human HCC cell line Li-7 (bio-54087) was obtained from Biobw (China), while the mouse HCC cell line (luciferase-labeled) Hepa 1-6-LUC (zl-056911) was obtained from Zhili Zhongte (Wuhan) Biotechnology Co., Ltd. (China). Cells were cultured in Dulbecco’s Modified Eagle Medium (DMEM) (11965092, Thermofisher, USA) supplemented with 100 U/mL penicillin, 100 μg/mL streptomycin, and 10% fetal bovine serum (Gibco, USA), and incubated in a CO_2_ incubator at 37 °C. Human splenic T cells (MZ, 4229, Zhejiang, China) and human CD8^+^ T cells were cultured using CTS™ OpTmizer™ T Cell Expansion SFM medium (A1048501, Thermo Fisher, USA). Human CD8^+^ T cells were isolated using Dynabeads™ CD8 Positive Isolation Kit (11333D, Thermo Fisher, USA) from T cells.

A 0.4 μm Transwell was used for co-culture of fibroblasts and cancer cells. Different secretory fibroblasts were seeded in the lower chamber of the Transwell (10^5^ cells/well), while the Li-7 HCC cell line was seeded in the upper chamber (5 × 10^4^ cells/well). In the Treg differentiation experiment, T cells were seeded in the upper chamber (10^5^ cells/well), and different IL34-secreting CAFs were seeded in the lower chamber (5 × 10^4^ cells/well). For validation of CD8^+^ T cell immunosuppression, CD8^+^ T cells were seeded in the upper chamber (5 × 10^4^ cells/well), and T cells treated with different secretory fibroblasts were seeded in the lower chamber (10^5^ cells/well), in medium containing Dynabeads™ Human T-Activator CD3/CD28 (11132D, Thermo Fisher, USA) and hrIL-2 (20 IU/mL, Thermo Fisher, PHC0021, USA). IL34-anti (PA5-95624, Thermo Fisher, USA) was added at a concentration of 50 ng/mL. Cells were collected 48 hours later for flow cytometric analysis to assess cytotoxicity of CD8^+^ T cells.

For the IL34 gradient experiment, CAFs were seeded in each well of a 12-well plate at a concentration of 5 × 104 cells, cultured under appropriate conditions, allowed to adhere for 24 hours, starved for 6 hours, and then either left unstimulated or stimulated with recombinant human IL34 (34-8684-63, Thermofisher, USA) for 6–48 hours. The resultant cells were analyzed for protein and gene expression by Western blotting or real-time PCR analysis^20,21^.

### Lentiviral transfection

The construction of lentiviral transfection was based on the specific cell lines, including IL34 knocked down human cancer-associated fibroblasts (sh-IL34) and their control cell line (sh-NC); IL34 overexpressed mouse cancer-associated fibroblasts (IL34-OC) and their control cell line (IL34-NC). The constructed plasmids containing a single luciferase reporter gene were co-transfected with the packaging plasmids into 293 T cells (CL-0469, ProMab Biotechnologies, China). After validation, amplification, and purification, packaged lentivirus was obtained. For lentivirus-mediated cell transfection, 5×10^5 cells were seeded in a 6-well plate and incubated until the confluence reached 70-90%. Then, the cells were transfected with an appropriate amount of packaged lentivirus (MOI = 10, working titer of approximately 5 × 10^6^ TU/mL) and 5 μg/mL polybrene (Merck, TR-1003, USA) in the culture medium. After 4 hours of transfection, an equal amount of medium was added to dilute polybrene. After 24 hours of transfection, the fresh medium was replaced. After 48 hours of transfection, the transfection efficiency was observed using the luciferase reporter gene, and stable cell lines were obtained by using 60 μg/mL ampicillin (Sangon Biotech, A100339, Shanghai, China) for resistance screening. The lentiviral infection procedure is as follows: logarithmically growing CAFs were prepared into a cell suspension with a concentration of 5×10^4^ cells/mL and seeded in a 6-well plate with 2 mL per well. The cells were incubated overnight at 37 °C, and then each well was added with a final concentration of 1 × 10^8^ TU/mL of silence or overexpression lentivirus. After 24 hours of infection, the infection efficiency was evaluated by qRT-PCR, and the cells with good effects were selected for subsequent experiments. Each experiment was repeated three times. The transfection sequence is shown in Supplementary Table 1.

### Flow cytometry analysis

Cell samples were washed with PBS and then resuspended for later use. Tissue samples were digested at 37 °C in PBS containing 0.8 mg/mL Collagenase IV (Merck, C4-BIOC, USA) for 30 minutes. The supernatant was collected after centrifugation at 850 × *g* for 10 minutes, followed by PBS washing. Dead cells were removed using Percoll (Merck, P1644, USA) according to the manufacturer’s instructions, and then the samples were resuspended. The concentrations were adjusted to a concentration of 1 × 10^7^ cells/mL in 100 µL of PBS for each sample. When intracellular antigens needed to be detected, cells were permeabilized for 5 minutes using 0.5% Tween 20 (Merck, P2287, USA) before incubation with the primary antibody. The samples were incubated with antibodies at 4 °C and then analyzed using a flow cytometer (BD Bioscience, BD LSRFortessa, USA). Anti-Human-IL34 (MA5-17098, Thermo Fisher, USA) and Vimentin (MA5-11883, Thermo Fisher, USA) were used for screening IL34+ CAFs. FITC-Mouse-anti-Human-Granzyme B (GzmB) (20 μL/test, BD Bioscience, 560211, USA) or Alexa Fluor® 488-Mouse-anti-Human-Ki67 (5 μL/test, BD Bioscience, 561165, USA) and APC-Mouse-anti-Human-CD8 (5 μL/test, BD Bioscience, 340584, USA) were used to evaluate the cytotoxicity of CD8^+^ T cells. Foxp3-PE (12-5773-82, Thermo Fisher, USA) and CD8-FITC (11-0081-82, Thermo Fisher, USA) were used to detect the proportion of Tregs cells. The obtained data were analyzed using BD FACSDiva software^22^.

### Immunofluorescence

The tested cells were seeded onto a 12-well cell culture plate for cell adhesion one day in advance. After the cells adhered to the plate, the culture medium in the 12-well plate was discarded, and the cell slides were washed three times with PBS for 5 minutes each time. The cells were fixed with 4% paraformaldehyde (PFA), permeabilized with 0.1% Triton X-100, and blocked with 10% normal donkey serum. Tumor tissue samples were fixed with 4% paraformaldehyde, dehydrated, made transparent, embedded in paraffin, and sectioned. For immunofluorescence staining, the sections were deparaffinized, rehydrated, and blocked with 2% BSA. Primary antibodies were added and incubated overnight at 4 °C. The primary antibodies used in the experiment included IL34 (MA5-17098, Thermofisher, 1:200, USA), CD31 (MA3100, Thermofisher, 1:200, USA), Cytokeratin (MA1-06312, Thermofisher, 1:200, USA), Vimentin (MA5-11883, Thermofisher, 1:250, USA), CSF1‐R (PA5-115557, Thermofisher, 1:100, USA), PTP-ζ (ab126497, Abcam, 1:100, UK), Foxp3 (13-5773-82, 1:50, Thermofisher, USA), and COL1A2 (PA5-50938, 1:200, Thermofisher, USA). On the second day, the sections were washed with PBS and incubated with goat anti-mouse IgG (A10551, Thermofisher, 1:200, USA) or goat anti-rabbit IgG (A-11008, Thermofisher, 1:500, USA) secondary antibodies at room temperature for 1 hour. After PBS wash, the nuclei were stained with DAPI (C1002, Beyotime, China) for 5 minutes, followed by three washes with PBS for 5 minutes each to remove excess DAPI. The cell slides were carefully removed from the cell culture plate using a bent needle and forceps and placed on a glass slide with anti-fluorescence quenching mounting medium (cell side down). They were then observed and photographed under a fluorescence microscope (FV-1000/ES, Olympus, Japan). The fluorescent coverage area was quantitatively analyzed under a 40x objective lens, and the average value was calculated from 6 fields of view in each group^22^.

### Immunohistochemical staining

The HCC or lung tissues from each mouse group were embedded and sectioned. The sections were then baked at a temperature of 60 °C for 20 minutes. Subsequently, they were immersed in xylene solution, with two changes of xylene for 15 minutes each. Afterward, they were treated with anhydrous ethanol for 5 minutes, followed by another 5 minutes with a change of anhydrous ethanol. The sections were then hydrated in 95% and 70% ethanol for 10 minutes each. Each slide was treated with 3% H_2_O_2_ and left to soak at room temperature for 10 minutes to block endogenous peroxidase activity. Citrate buffer was added, and the sections were microwaved for 3 minutes. Antigen retrieval solution was then added and left to stand at room temperature for 10 minutes, followed by three washes with PBS. The sections were incubated with blocking solution consisting of normal goat serum (E510009, Shanghai Biochemical Engineering Co., Ltd.) for 20 minutes at room temperature. The diluted Foxp3 primary antibody (13-5773-82, 1:50, Thermofisher, USA) was then added and incubated overnight at 4 °C. After three washes in PBS, the sections were incubated with goat anti-rabbit IgG secondary antibody (ab6721, 1:500, Abcam, UK) for 30 minutes. Following another wash with PBS, the sections were subjected to DAB chromogenic reagent (Sigma, USA) with drops of the A, B, and C solutions added to the specimens. The sections were then stained with hematoxylin for 30 seconds, dehydrated in a series of ethanol (70%, 80%, 90%, and 95%) and two changes of xylene for 2 minutes each. Finally, they were sealed with neutral resin and observed under a upright microscope (BX63, Olympus, Japan). The immunohistochemistry method employed the Aperio Scanscope System (Vista, CA) to measure the area of positive protein expression. The cell area was calculated as the product of the total protein-positive area and the corresponding tissue weight^23^.

### H&E staining

The lung tissues of mice from each group were collected and fixed in 10% neutral formalin. They were then embedded in paraffin for sectioning, and dewaxed with xylene. Subsequently, the sections were stained with hematoxylin, washed with distilled water, immersed in 95% ethanol, stained with eosin, dehydrated using a gradient of ethanol and xylene, air-dried, and finally observed under an optical microscope^24^.

### In situ mouse HCC model construction

Thirty-six 6-week-old C57BL/6N mice were purchased from Beijing Vital River Laboratory Animal Technology Co., Ltd. (Beijing, China). The mice were housed in standard cages under constant room temperature (23 ± 1 °C) with a 12-hour light/dark cycle and 60%-65% humidity. They had ad libitum access to food and water and were acclimatized for one week prior to the experiments. The experimental procedures and animal usage were approved by the Animal Ethics Committee of Shanghai Pudong Hospital.

For the establishment of the in situ mouse HCC model, Hepa 1-6-LUC cells in logarithmic growth phase were cultured under appropriate conditions. The cells were suspended in PBS at a concentration of 1 × 10^6^/ml. The mice were anesthetized by intraperitoneal injection of 1% pentobarbital (60 mg/kg). After the anesthesia took effect, the mice were dissected, and their livers were exposed. A mixture of 50 μl cell suspension and matrigel matrix (354234, Corning, China) was implanted under the capsule of the left hepatic lobe. The incisions were closed with sutures or clips. The entire procedure was performed under sterile conditions^25^. No analgesics were administered to the mice during the experiment.

The aim of the in situ mouse HCC model was to implant a mixture of conventional resuscitated Hepa 1-6-LUC cells and mouse CAFs treated with lentivirus into the mice after culturing them under appropriate conditions. The implantation procedure was the same as described above. During tumor growth, Foxp3-anti (14-4774-82, Thermo Fisher, USA) was intravenously injected on days 1, 4, 7, and 10. The injection dose was 1.2 mg/kg. Two weeks later, tumor volume was recorded using the formula V = 0.5 × L × W^2^ (where V is tumor volume, L is the longest tumor length, and W is the shortest tumor length). Tumor tissue samples were collected for subsequent experiments. The mice were anesthetized via intraperitoneal injection of 1% pentobarbital (60 mg/kg) for tissue collection. After the anesthesia took effect, the mice were dissected by opening their abdominal cavity to expose the liver. The tumors were then removed, and euthanasia was performed using the cervical dislocation method. The specific groups were as follows: HCC cells mixed with CAFs treated with control (IL34-NC+IgG), HCC cells mixed with CAFs treated with overexpression (IL34-OC+IgG), and HCC cells mixed with CAFs treated with overexpression and Foxp3-anti (IL34-OC+Foxp3-anti).

### RT-qPCR

Total RNA from tissues and cells was extracted using Trizol (Thermo Fisher, 16096020, USA). cDNA was synthesized by reverse transcription using the reverse transcription kit (Takara, RR047A, Japan). The reaction mixture was prepared using SYBR® Premix Ex TaqTM II kit (Takara, DRR081, Japan) and real-time quantitative PCR (RT-qPCR) was performed using the ABI 7500 real-time fluorescent PCR system (Thermo Fisher, USA). β-actin was used as the reference gene. The PCR program consisted of an initial denaturation at 95 °C for 30 s, followed by 40 cycles of denaturation at 95 °C for 5 s, annealing at 60 °C for 30 s, and extension at 95 °C for 15 s, 60 °C for 60 s, and 90 °C for 15 s, followed by amplification curve analysis. Each RT-qPCR was performed in triplicate. The primer sequences are shown in Supplementary Table 2. The relative expression of the target gene in the experimental group compared to the control group was calculated using the 2^-ΔΔCt^ method, where ΔΔCt = ΔCt experimental group - ΔCt control group, and ΔCt = Ct_target gene_ - Ct_reference gene_. Ct represents the amplification cycle number when the real-time fluorescence intensity reaches the threshold^26^. The experiment was repeated three times.

### Western blot

Cell and tissue total protein was extracted using the Bestbio protein extraction kit (BB3101, Shanghai, China). Protein concentration was determined using the BCA assay kit (Bi Yun Tian, P0012S, Shanghai, China). A 10% SDS-PAGE gel (Bi Yun Tian, P0012A, Shanghai, China) was prepared, and each well was loaded with 50 μg of protein sample. Electrophoresis was performed at a constant voltage of 80-120 V for 2 hours. Wet transfer at a constant current of 250 mA was conducted for 90 minutes to transfer the proteins onto a PVDF membrane (Merck, IPVH00010, Germany). The PVDF membrane was then blocked with TBST containing 5% skim milk at room temperature for 2 hours, followed by three washes with TBST for 10 minutes each. The primary antibody incubation was carried out overnight at 4 °C (Supplementary Table 3 for antibody information), followed by three washes with TBST for 10 minutes each. The PVDF membrane was then incubated with Goat anti-rabbit IgG (1:2000, Abcam, ab6721, UK) or Goat anti-mouse IgG (1:2000, Abcam, ab6789, UK) at room temperature for 1 hour, followed by three washes with PBST for 10 minutes each. The ECL detection kit (Bi Yun Tian, P0018FS, Shanghai, China) was used for signal detection, and exposure and development were performed in a dark box^27^. Each experiment was repeated three times for each sample. All blots were derived from the same experiment and were processed in parallel.

### CCK8 and plate cloning experiments

The CCK-8 assay kit (Beyotime, C0037, Shanghai, China) was used to evaluate cell proliferation. Log-phase cells were adjusted to a concentration of 5 × 10^4^ cells/mL in DMEM-H medium supplemented with 10% FBS. Subsequently, 100 μL of cell suspension was added to each well of a 96-well culture plate and incubated in a CO_2_ incubator for 48 h. After discarding the supernatant, 10 μL of CCK-8 reagent was added to each well and incubated for an additional 2 h at 37 °C. The absorbance at 450 nm was measured using a Multiskan FC microplate reader (Thermo Fisher, 51119080, USA). Each group was assessed in triplicate, and the average value was reported^28^.

For the colony formation experiment, cells in the logarithmic growth phase were dissociated using standard digestion methods to obtain a single-cell suspension with a viability greater than 95%. The cells were counted and diluted with culture medium to an appropriate concentration. Next, 5 mL of cell suspension, containing approximately 100 cells per dish, was seeded in a 60 mm Petri dish. The dish was gently swirled in a cross direction to ensure even cell dispersion. The culture dishes were then incubated at 37 °C with 5% CO_2_ for 2–3 weeks. When visible colonies formed, the culture was terminated. The culture medium was removed, and the dishes were rinsed twice with PBS solution and air-dried. The cells were fixed in methanol for 15 minutes, followed by air-drying after removing the methanol. After staining with Giemsa solution for 10 minutes, excess stain was rinsed off under slow-running water, and air-dried. The number of colonies containing more than 10 cells was counted either using the naked eye or under a low-power microscope. The colony formation rate was calculated using the formula: colony formation rate = (number of colonies/number of seeded cells) × 100%^29^.

### Scratch test

The technique involved for this experiment begins with using a marking pen to draw evenly spaced horizontal lines on the back of a 6-well plate, with a gap of 1 cm between each line, traversing across the holes. Each hole is then inoculated with approximately 5 × 10^5^ cells, and the plate is cultured conventionally until confluency reaches 100%. At that point, a scratch is made on the cell layer by holding a pipette tip perpendicular to the cell plane and dragging it along the line on the back of the plate. After completing the scratch, the cell layer is washed three times with sterile PBS to remove non-adherent cells, making the gap left by the scratch clearly visible. Subsequently, fresh serum-free culture medium is replaced, and the cells are incubated at 37 °C 5% CO_2_. After 24 hours, the width of the scratch is observed and measured under a microscope, and the results are documented by taking photographs^30^. Migration analysis is performed using Image J software.

### Transwell assay

To log-phase cells, they were washed once with PBS and serum-free medium after digestion. The cells were suspended in serum-free medium, counted, and adjusted to a concentration of 2 × 105/mL. For invasion experiments, Matrigel gel (Corning, 356237, USA) was thawed at 4 °C overnight after being removed from –20 °C freezer. Matrigel gel was then diluted to 300 μL/mL with serum-free cell culture medium at 4 °C. 100 μL of the diluted gel was evenly spread onto the surface of a PET membrane in a cell culture well. The well plate was gently placed into a 24-well plate at 37 °C for approximately 3 hours. After removing it from the laminar flow hood, it was left to dry overnight. For migration experiments, Matrigel gel coating was not needed. 800 μL of regular culture medium containing 10% serum was added to the lower chamber (i.e. the bottom of the 24-well plate), and 150 μL of cell suspension was added to the upper chamber. The plate was then incubated in a CO_2_ incubator for 24 hours. After discarding the residual liquid in the upper and lower chambers, any remaining gel and non-invading cells in the upper chamber were gently wiped off with a clean cotton swab. 0.5 mL of 4% polyformaldehyde was added for fixation, followed by removal after 30 minutes. Subsequently, 0.5 mL of 0.1% crystal violet was added for staining, and after a 20-minute incubation, it was removed. The PET membrane was washed three times with PBS, and then examined under a microscope. The number of cells on the underside of the PET membrane was calculated by counting 5 fields in the middle and four corners, and taking the average^30^.

### Statistical analysis

Our study utilized R language, version 4.2.1 (https://www.r-project.org/), for data analysis. The RStudio integrated development environment, version 4.2.1 (https://rstudio.com/), was employed for R language compilation. Perl language, version 5.30.0 (https://www.perl.org/), was used for file processing. Cytoscape, version 3.7.2 (https://cytoscape.org/), and Origin statistical software, version 2021, were utilized for data visualization and statistical analysis, respectively. For quantitative data, means ± standard deviations were reported. Between-group comparisons were conducted using independent sample t-test. One-way analysis of variance (ANOVA) was employed for comparing data among different groups, while two-way ANOVA was used for comparing data among different time points within groups. Post-hoc tests were performed using the Bonferroni method. A *p*-value of <0.05 was considered statistically significant.

### Reporting summary

Further information on research design is available in the Nature Research Reporting Summary linked to this article.

### Supplementary information

Supplementary information file

REPORTING SUMMARY

## Data Availability

The data that supports the findings of this study are available on request from the corresponding author. All the Raw data is available at Figshare: 10.6084/m9.figshare.24433423.
