# Supplementary material for: Single-cell transcriptome sequencing reveals spatial distribution of IL34+ cancer-associated fibroblasts in hepatocellular carcinoma tumor microenvironment
Source: NPJ Precis Oncol. 2023 Dec 11;7:133. doi: 10.1038/s41698-023-00483-9 (PMC10713639; doi:10.1038/s41698-023-00483-9)
Supplement: Supplementary file 1 — Supplementary information file [file 41698_2023_483_MOESM1_ESM.pdf]

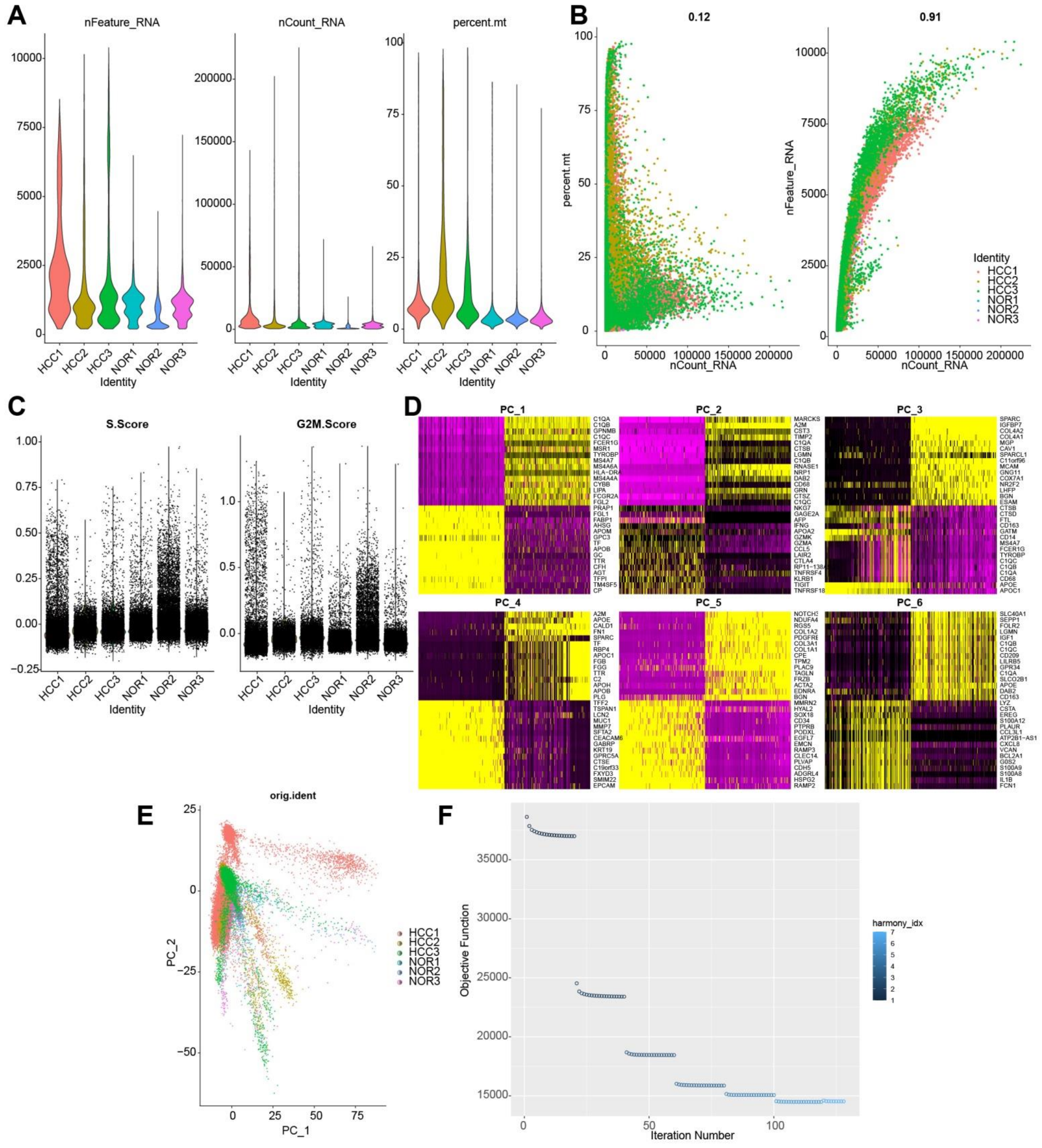

Supplementary Figure 1. Quality control and PCA dimension reduction of scRNA-seq data

Note: (A) Violin plots showing the number of genes per cell (nFeature_RNA), the number of mRNA molecules (nCount_RNA), and the percentage of mitochondrial genes (percent.mt) in scRNA-seq data. (B) Scatter plots illustrating the correlation between filtered data nCount_RNA and percent.mt, as well as between nCount_RNA and nFeature_RNA. (C) Cell cycle states of each cell in scRNA-seq data, with

S.Score representing the S phase and G2M.Score representing the G2M phase. (D) Heatmaps displaying the top 20 significantly correlated gene expressions in PC_1 – PC_6 of the PCA analysis, where yellow indicates upregulated expression and purple indicates downregulated expression. (E) Distribution of cells in PC_1 and PC_2 before batch correction, with each point representing a cell. (F) Batch correction process graph using Harmony, with the x-axis denoting the number of iterative interactions.

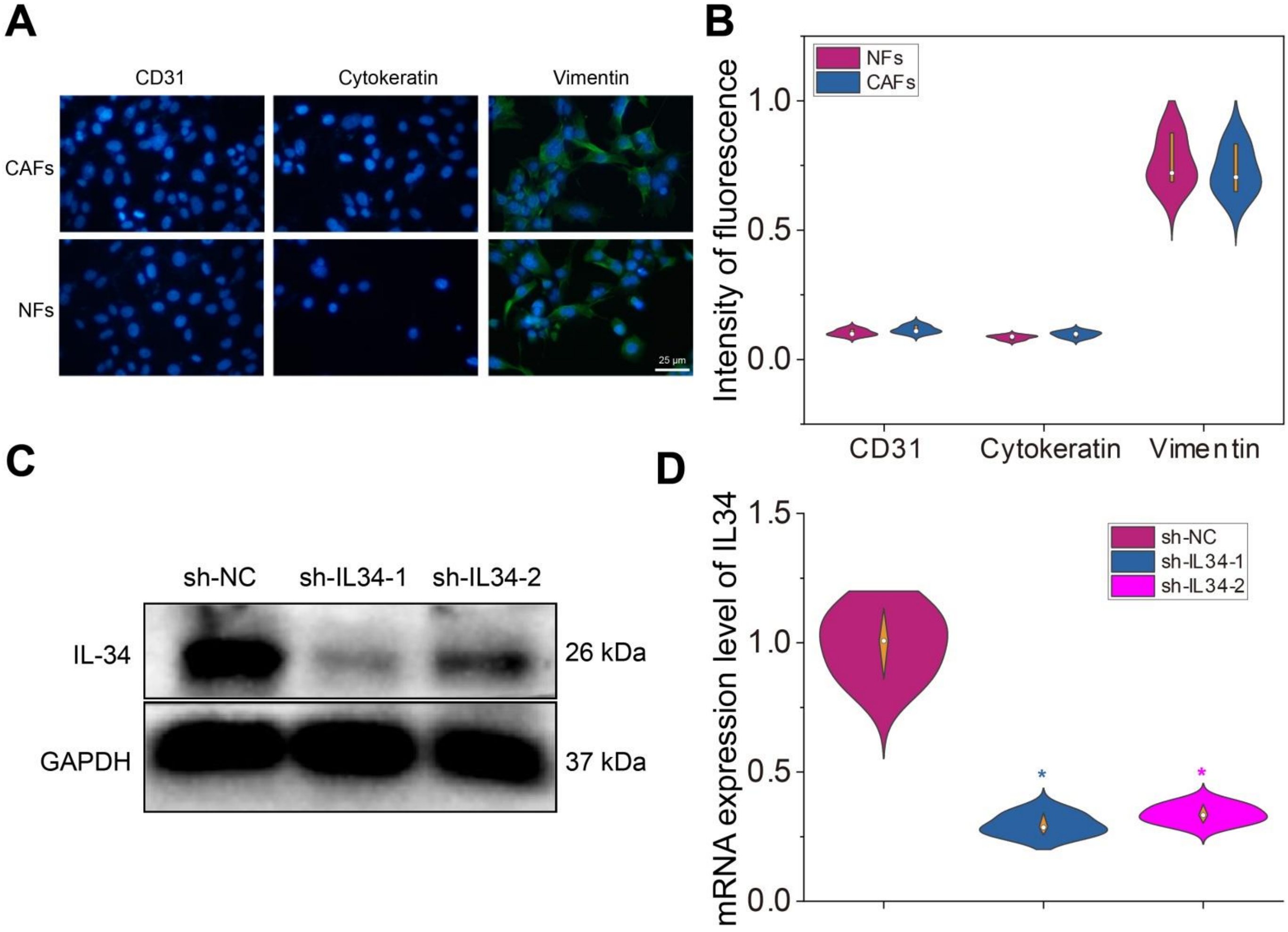

Supplementary Figure 2. Validation of the quality and silencing effect of fibroblasts

Note: (A) Immunofluorescence detection of the expression of endothelial cell marker protein CD31, epithelial cell marker protein Cytokeratin, and fibroblast marker protein Vimentin. CD31, Cytokeratin, and Vimentin are shown in green, DAPI in blue, and the scale bar is 75 µm. (B) Immunofluorescence intensity statistics graph. (C) Validation of the silencing effect of IL34 in tumor fibroblasts through Western Blot. (D) Validation of the silencing effect of IL34 in tumor fibroblasts through RT-qPCR. * represents $P < 0.05$ compared to the sh-NC group. All cell experiments were repeated three times.

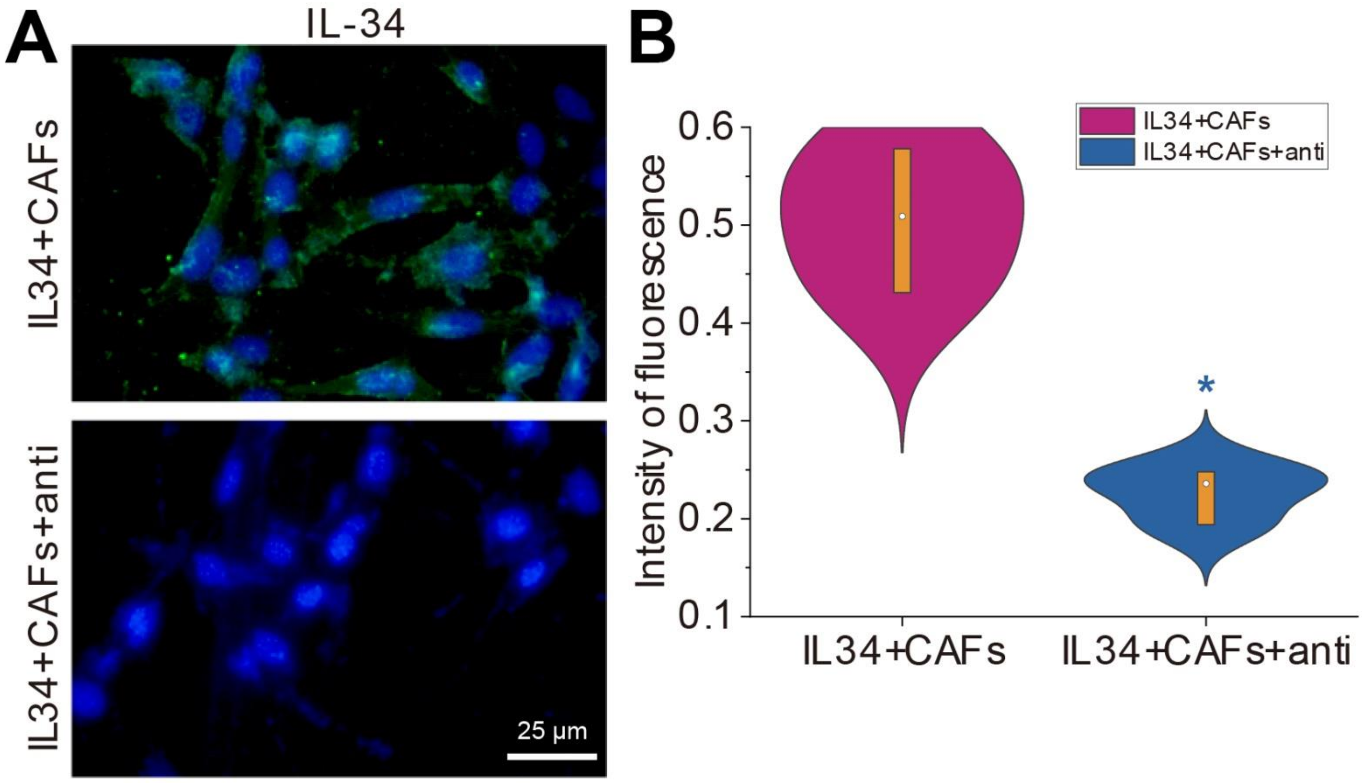

Supplementary Figure 3. Validation of IL34-blocking effect

Note: (A) Immunofluorescence staining showing the expression of IL34 in IL34+ CAFs after antibody treatment. IL34 (green), DAPI (blue), scale bar: 75 μm. (B) Bar graph representing fluorescence intensity. * denotes a significant difference (P < 0.05) compared to the IL34+ CAFs group. All cellular experiments were performed in triplicate.

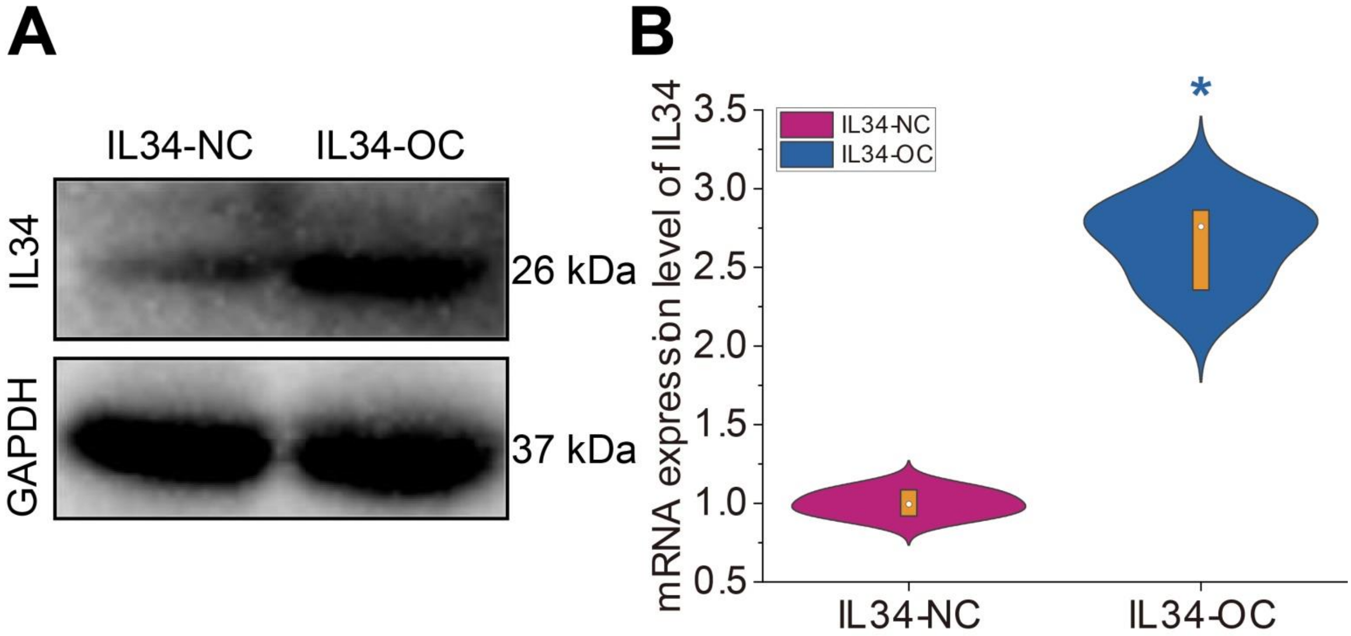

Supplementary Figure 4. Validation of overexpression effects

Note: (A) Western blot was performed to examine the expression of IL34 in CAFs after lentivirus treatment; (B) RT-qPCR was conducted to detect the expression of IL34 in CAFs after lentivirus treatment. * indicates a significant difference compared to the IL34-NC group at a *p*-value of less than 0.05. All cell experiments were repeated three times.

WB images

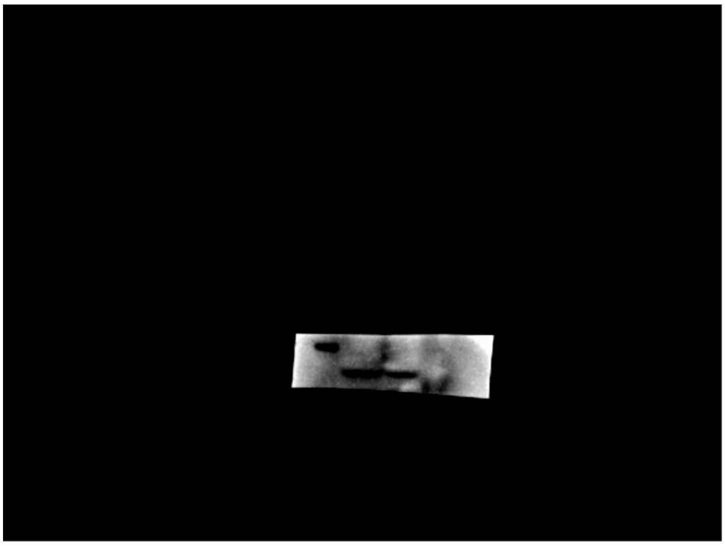

Supplementary Figure 5

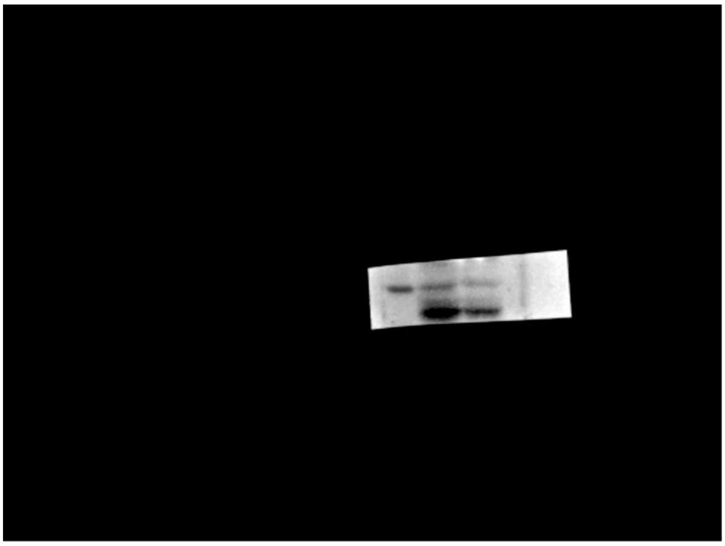

Supplementary Figure 6

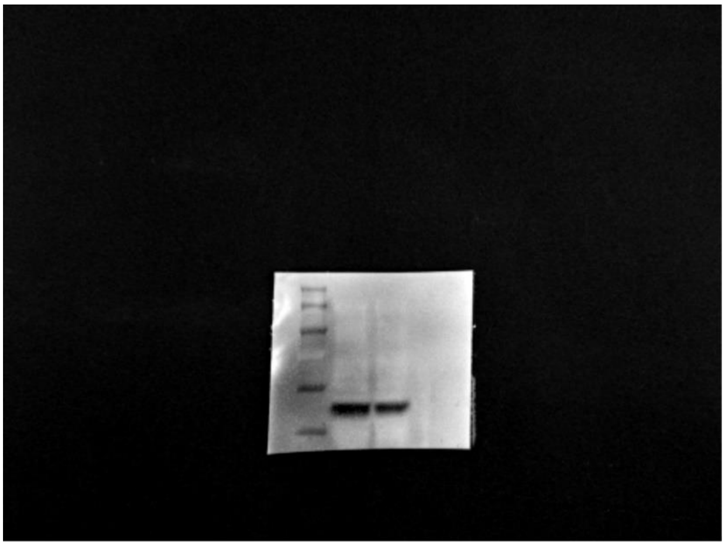

Supplementary Figure 7

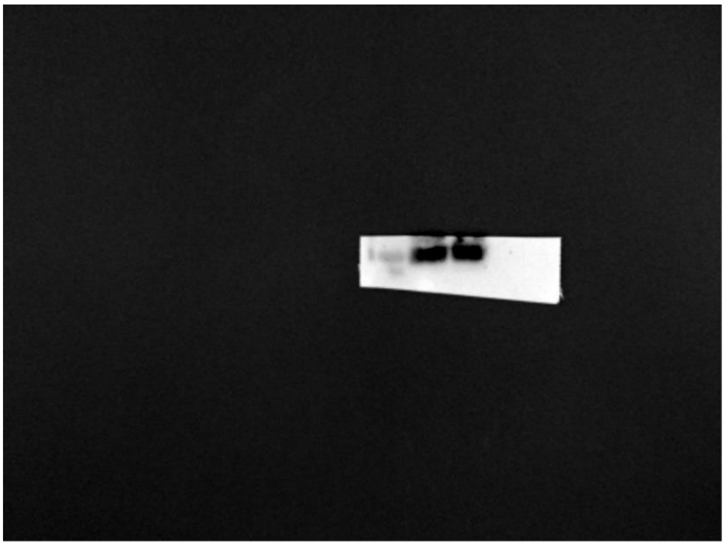

Supplementary Figure 8

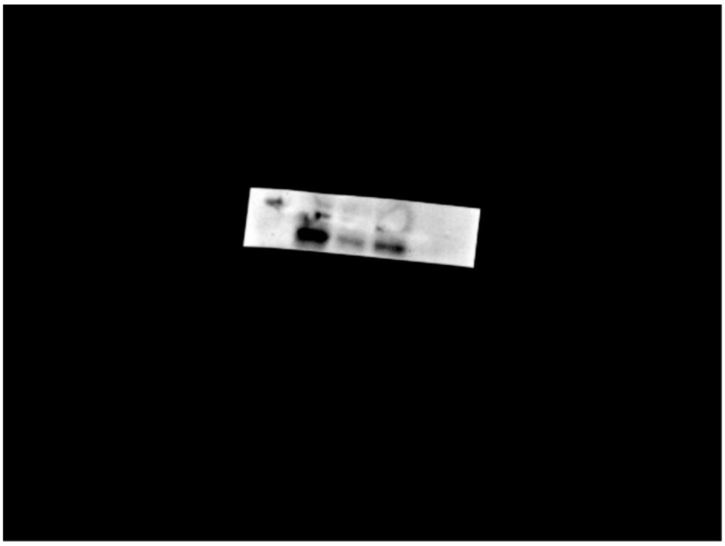

Supplementary Figure 9

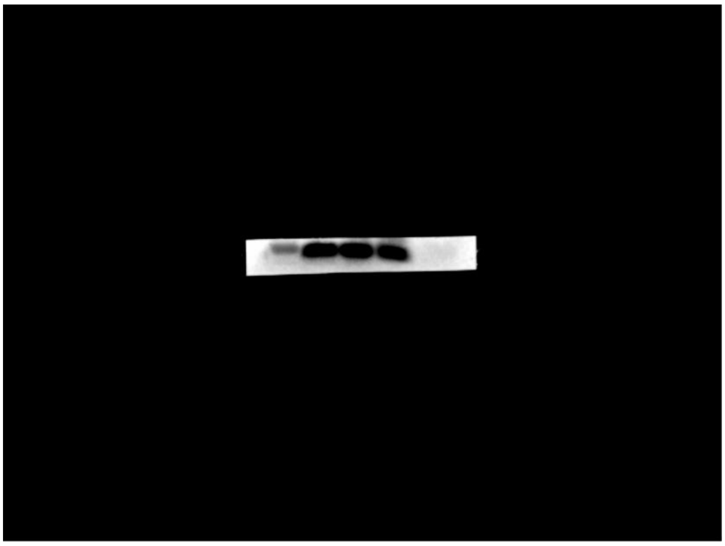

Supplementary Figure 10

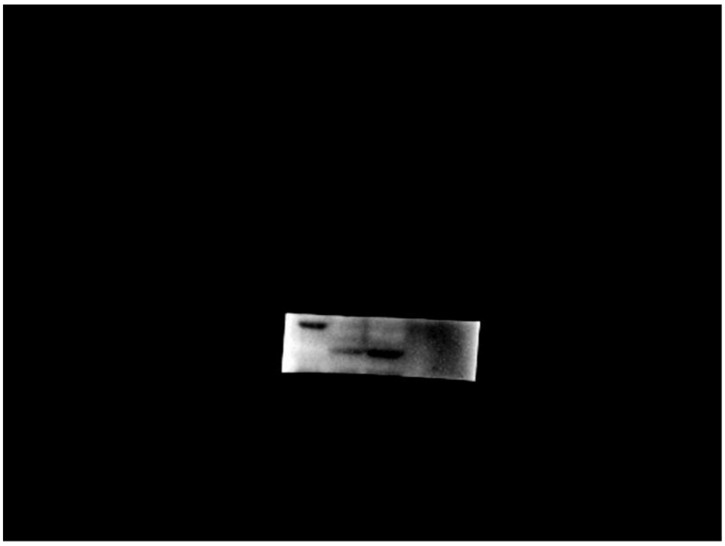

Supplementary Figure 11

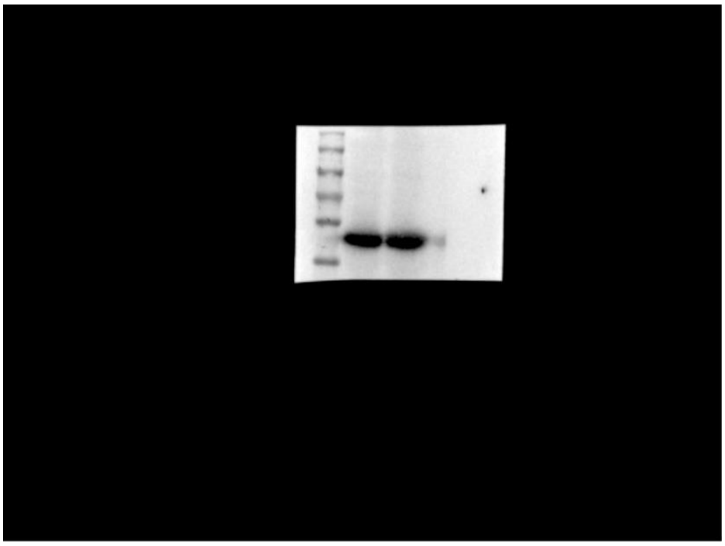

Supplementary Figure 12

Supplementary Table 1. Primer sequences for lentiviral transfection

Name	Sequences
sh-IL34-1 (human)	5'-GCCGACTTCAGTACATGAAAC-3'
sh-IL34-2 (human)	5'-GACTTCAGTACATGAAACACT-3'
sh-NC	5'-CCTAAGGTTAAGTCGCCCTCG-3'

Supplementary Table 2. RT-qPCR primer sequences

Gene	Sequences (5'-3')
IL34 (Human)	Forward: ACAGGAGCCGACTTCAGTAC
	Reverse: ACCAAGACCCACAGATACCG
CSF1-R (Human)	Forward: CTGCTCAACTTTCTGCGAAG
	Reverse: CTCATCTCCACATAGGTGTC
PTP-ζ (Human)	Forward: ACTAACCGATCCCCAACAAG
	Reverse: CCACACATTTCCCTCCATAG
α-SMA (Human)	Forward: GTTCCGCTCCTCTCTCCAAC
	Reverse: GTGCGGACAGGAATTGAAGC
FAP (Human)	Forward: AGAACCATGCTTTGGAGATACT
	Reverse: TTTACTCCCAACAGGCGACC
Vimentin (Human)	Forward: GGACCAGCTAACCAACGACA
	Reverse: AAGGTCAAGACGTGCCAGAG
β-actin (Human)	Forward: GAGAAAATCTGGCACCACACC
	Reverse: GGATAGCACAGCCTGGATAGCAA

Supplementary Table 3. Western blot antibody information

Target name	Manufacturers	Item No.	Dilution ratio
IL34 (Human/Mouse)	Thermofisher	PA5-95624	1:1000
α-SMA (Human/Mouse)	Abbkine	ABM0052	1:10,000
FAP (Human/Mouse)	Thermofisher	PA5-51057	1:1000
Vimentin (Human/Mouse)	Thermofisher	MA5-11883	1:1000
GAPDH (Human/Mouse)	Thermofisher	MA5-15738	1:1000

Notes: Thermofisher, USA; Abbkine, USA.
