# Supplementary material for: Single-cell transcriptome sequencing reveals spatial distribution of IL34+ cancer-associated fibroblasts in hepatocellular carcinoma tumor microenvironment
Source: NPJ Precis Oncol. 2023 Dec 11;7:133. doi: 10.1038/s41698-023-00483-9 (PMC10713639; doi:10.1038/s41698-023-00483-9)
Supplement: Supplementary file 2 — REPORTING SUMMARY [file 41698_2023_483_MOESM2_ESM.pdf]

nature portfolio

Corresponding author(s): Xiaoliang Wang

Last updated by author(s): Oct 24, 2023

Reporting Summary

Nature Portfolio wishes to improve the reproducibility of the work that we publish. This form provides structure for consistency and transparency in reporting. For further information on Nature Portfolio policies, see our Editorial Policies and the Editorial Policy Checklist.

Statistics

For all statistical analyses, confirm that the following items are present in the figure legend, table legend, main text, or Methods section.

n/a	Confirmed	
☐	☒	The exact sample size (*n*) for each experimental group/condition, given as a discrete number and unit of measurement
☐	☒	A statement on whether measurements were taken from distinct samples or whether the same sample was measured repeatedly
☐	☒	The statistical test(s) used AND whether they are one- or two-sided<br>*Only common tests should be described solely by name; describe more complex techniques in the Methods section.*
☐	☒	A description of all covariates tested
☐	☒	A description of any assumptions or corrections, such as tests of normality and adjustment for multiple comparisons
☐	☒	A full description of the statistical parameters including central tendency (e.g. means) or other basic estimates (e.g. regression coefficient) AND variation (e.g. standard deviation) or associated estimates of uncertainty (e.g. confidence intervals)
☐	☒	For null hypothesis testing, the test statistic (e.g. *F*, *t*, *r*) with confidence intervals, effect sizes, degrees of freedom and *P* value noted<br>*Give P values as exact values whenever suitable.*
☐	☒	For Bayesian analysis, information on the choice of priors and Markov chain Monte Carlo settings
☐	☒	For hierarchical and complex designs, identification of the appropriate level for tests and full reporting of outcomes
☐	☒	Estimates of effect sizes (e.g. Cohen's *d*, Pearson's *r*), indicating how they were calculated

Our web collection on statistics for biologists contains articles on many of the points above.

Software and code

Policy information about availability of computer code

Data collection	https://doi.org/10.6084/m9.figshare.24433423
Data analysis	https://doi.org/10.6084/m9.figshare.24433423

For manuscripts utilizing custom algorithms or software that are central to the research but not yet described in published literature, software must be made available to editors and reviewers. We strongly encourage code deposition in a community repository (e.g. GitHub). See the Nature Portfolio guidelines for submitting code & software for further information.

Data

Policy information about availability of data

All manuscripts must include a data availability statement. This statement should provide the following information, where applicable:

- Accession codes, unique identifiers, or web links for publicly available datasets
- A description of any restrictions on data availability
- For clinical datasets or third party data, please ensure that the statement adheres to our policy

Provide your data availability statement here.

Research involving human participants, their data, or biological material

Policy information about studies with human participants or human data. See also policy information about sex, gender (identity/presentation), and sexual orientation and race, ethnicity and racism.

Reporting on sex and gender

Our study utilized tumor samples and non-tumor tissues obtained from 9 patients who underwent HCC treatment at our hospital between January 2019 and January 2021. Prior to surgery, all patients did not receive radiotherapy or chemotherapy. All subjects signed written informed consent before participating in our study. The study has been approved by Ethics Committee of Shanghai Pudong Hospital (QWJWLX-01) and complies with the Declaration of Helsinki. Collected tumor and non-tumor tissues were washed twice with pre-cooled PBS solution containing 2% gentamicin (15140148, Thermofisher, USA) in order to remove blood clots and necrotic tissue from the tissue surface. After cutting the tissues into small pieces with scissors, they were added to a solution of 0.1% type IV collagenase (17104019, Thermofisher, USA) containing 10% fetal bovine serum (16140089, Thermofisher, USA). This mixture was then transferred to a centrifuge tube and digested at a constant temperature of 37°C for 30-40 minutes on a shaker. The digestion medium, along with any remaining tissues, was passed through a 200-mesh sieve, and the filtrate was centrifuged at 4°C for 5 minutes (50 ×g) and the supernatant was discarded. The pellet was resuspended in complete DMEM medium (11965092, Thermofisher, USA) and washed twice. Red blood cells were removed using red blood cell lysis buffer (C3702-120 ml; Beyotime, China), and the cell density was adjusted to 106/ml. Based on the differences in fibroblast growth rate and adhesion ability compared to other cells, the cell suspension was added to the first well of a 6-well plate and allowed to incubate for 20 minutes. The cells adhering to the plate were predominantly fibroblasts, and the supernatant was aspirated and transferred to the second well for a further 20 minutes of incubation. This process was repeated, and the cells adhering to the two wells were agitated with DMEM containing 10% fetal bovine serum, and then cultured and passaged in a humidified incubator at 37°C and 5% CO_2, with media changes every 3 days. Immunofluorescence detection was performed to identify fibroblast marker protein expression in the obtained fibroblasts. Mouse CAFs were obtained by isolating tumor tissues from an in situ mouse HCC model (two weeks).

Reporting on race, ethnicity, or other socially relevant groupings

Our study utilized tumor samples and non-tumor tissues obtained from 9 patients who underwent HCC treatment at our hospital between January 2019 and January 2021. Prior to surgery, all patients did not receive radiotherapy or chemotherapy. All subjects signed written informed consent before participating in our study. The study has been approved by Ethics Committee of Shanghai Pudong Hospital (QWJWLX-01) and complies with the Declaration of Helsinki. Collected tumor and non-tumor tissues were washed twice with pre-cooled PBS solution containing 2% gentamicin (15140148, Thermofisher, USA) in order to remove blood clots and necrotic tissue from the tissue surface. After cutting the tissues into small pieces with scissors, they were added to a solution of 0.1% type IV collagenase (17104019, Thermofisher, USA) containing 10% fetal bovine serum (16140089, Thermofisher, USA). This mixture was then transferred to a centrifuge tube and digested at a constant temperature of 37°C for 30-40 minutes on a shaker. The digestion medium, along with any remaining tissues, was passed through a 200-mesh sieve, and the filtrate was centrifuged at 4°C for 5 minutes (50 ×g) and the supernatant was discarded. The pellet was resuspended in complete DMEM medium (11965092, Thermofisher, USA) and washed twice. Red blood cells were removed using red blood cell lysis buffer (C3702-120 ml; Beyotime, China), and the cell density was adjusted to 106/ml. Based on the differences in fibroblast growth rate and adhesion ability compared to other cells, the cell suspension was added to the first well of a 6-well plate and allowed to incubate for 20 minutes. The cells adhering to the plate were predominantly fibroblasts, and the supernatant was aspirated and transferred to the second well for a further 20 minutes of incubation. This process was repeated, and the cells adhering to the two wells were agitated with DMEM containing 10% fetal bovine serum, and then cultured and passaged in a humidified incubator at 37°C and 5% CO_2, with media changes every 3 days. Immunofluorescence detection was performed to identify fibroblast marker protein expression in the obtained fibroblasts. Mouse CAFs were obtained by isolating tumor tissues from an in situ mouse HCC model (two weeks).

Population characteristics

Our study utilized tumor samples and non-tumor tissues obtained from 9 patients who underwent HCC treatment at our hospital between January 2019 and January 2021. Prior to surgery, all patients did not receive radiotherapy or chemotherapy. All subjects signed written informed consent before participating in our study. The study has been approved by Ethics Committee of Shanghai Pudong Hospital (QWJWLX-01) and complies with the Declaration of Helsinki. Collected tumor and non-tumor tissues were washed twice with pre-cooled PBS solution containing 2% gentamicin (15140148, Thermofisher, USA) in order to remove blood clots and necrotic tissue from the tissue surface. After cutting the tissues into small pieces with scissors, they were added to a solution of 0.1% type IV collagenase (17104019, Thermofisher, USA) containing 10% fetal bovine serum (16140089, Thermofisher, USA). This mixture was then transferred to a centrifuge tube and digested at a constant temperature of 37°C for 30-40 minutes on a shaker. The digestion medium, along with any remaining tissues, was passed through a 200-mesh sieve, and the filtrate was centrifuged at 4°C for 5 minutes (50 ×g) and the supernatant was discarded. The pellet was resuspended in complete DMEM medium (11965092, Thermofisher, USA) and washed twice. Red blood cells were removed using red blood cell lysis buffer (C3702-120 ml; Beyotime, China), and the cell density was adjusted to 106/ml. Based on the differences in fibroblast growth rate and adhesion ability compared to other cells, the cell suspension was added to the first well of a 6-well plate and allowed to incubate for 20 minutes. The cells adhering to the plate were predominantly fibroblasts, and the supernatant was aspirated and transferred to the second well for a further 20 minutes of incubation. This process was repeated, and the cells adhering to the two wells were agitated with DMEM containing 10% fetal bovine serum, and then cultured and passaged in a humidified incubator at 37°C and 5% CO_2, with media changes every 3 days. Immunofluorescence detection was performed to identify fibroblast marker protein expression in the obtained fibroblasts. Mouse CAFs were obtained by isolating tumor tissues from an in situ mouse HCC model (two weeks).

Recruitment

Our study utilized tumor samples and non-tumor tissues obtained from 9 patients who underwent HCC treatment at our hospital between January 2019 and January 2021. Prior to surgery, all patients did not receive radiotherapy or chemotherapy. All subjects signed written informed consent before participating in our study. The study has been approved

by Ethics Committee of Shanghai Pudong Hospital (QWJWLX-01) and complies with the Declaration of Helsinki. Collected tumor and non-tumor tissues were washed twice with pre-cooled PBS solution containing 2% gentamicin (15140148, Thermofisher, USA) in order to remove blood clots and necrotic tissue from the tissue surface. After cutting the tissues into small pieces with scissors, they were added to a solution of 0.1% type IV collagenase (17104019, Thermofisher, USA) containing 10% fetal bovine serum (16140089, Thermofisher, USA). This mixture was then transferred to a centrifuge tube and digested at a constant temperature of 37°C for 30-40 minutes on a shaker. The digestion medium, along with any remaining tissues, was passed through a 200-mesh sieve, and the filtrate was centrifuged at 4°C for 5 minutes (50 ×g) and the supernatant was discarded. The pellet was resuspended in complete DMEM medium (11965092, Thermofisher, USA) and washed twice. Red blood cells were removed using red blood cell lysis buffer (C3702-120 ml; Beyotime, China), and the cell density was adjusted to 106/ml. Based on the differences in fibroblast growth rate and adhesion ability compared to other cells, the cell suspension was added to the first well of a 6-well plate and allowed to incubate for 20 minutes. The cells adhering to the plate were predominantly fibroblasts, and the supernatant was aspirated and transferred to the second well for a further 20 minutes of incubation. This process was repeated, and the cells adhering to the two wells were agitated with DMEM containing 10% fetal bovine serum, and then cultured and passaged in a humidified incubator at 37°C and 5% CO2, with media changes every 3 days. Immunofluorescence detection was performed to identify fibroblast marker protein expression in the obtained fibroblasts. Mouse CAFs were obtained by isolating tumor tissues from an in situ mouse HCC model (two weeks).

Ethics oversight

The study has been approved by Ethics Committee of Shanghai Pudong Hospital (QWJWLX-01) and complies with the Declaration of Helsinki.

Note that full information on the approval of the study protocol must also be provided in the manuscript.

Field-specific reporting

Please select the one below that is the best fit for your research. If you are not sure, read the appropriate sections before making your selection.

☒ Life sciences ☐ Behavioural & social sciences ☐ Ecological, evolutionary & environmental sciences

For a reference copy of the document with all sections, see nature.com/documents/nr-reporting-summary-flat.pdf

Life sciences study design

All studies must disclose on these points even when the disclosure is negative.

Sample size

Our study utilized tumor samples and non-tumor tissues obtained from 9 patients who underwent HCC treatment at our hospital between January 2019 and January 2021. Prior to surgery, all patients did not receive radiotherapy or chemotherapy. All subjects signed written informed consent before participating in our study.

Data exclusions

Our study utilized tumor samples and non-tumor tissues obtained from 9 patients who underwent HCC treatment at our hospital between January 2019 and January 2021. Prior to surgery, all patients did not receive radiotherapy or chemotherapy. All subjects signed written informed consent before participating in our study.

Replication

Our study utilized tumor samples and non-tumor tissues obtained from 9 patients who underwent HCC treatment at our hospital between January 2019 and January 2021. Prior to surgery, all patients did not receive radiotherapy or chemotherapy. All subjects signed written informed consent before participating in our study.

Randomization

Our study utilized tumor samples and non-tumor tissues obtained from 9 patients who underwent HCC treatment at our hospital between January 2019 and January 2021. Prior to surgery, all patients did not receive radiotherapy or chemotherapy. All subjects signed written informed consent before participating in our study.

Blinding

Our study utilized tumor samples and non-tumor tissues obtained from 9 patients who underwent HCC treatment at our hospital between January 2019 and January 2021. Prior to surgery, all patients did not receive radiotherapy or chemotherapy. All subjects signed written informed consent before participating in our study.

Reporting for specific materials, systems and methods

We require information from authors about some types of materials, experimental systems and methods used in many studies. Here, indicate whether each material, system or method listed is relevant to your study. If you are not sure if a list item applies to your research, read the appropriate section before selecting a response.

Materials & experimental systems

n/a	Involved in the study
☐	☒ Antibodies
☒	☐ Eukaryotic cell lines
☒	☐ Palaeontology and archaeology
☐	☒ Animals and other organisms
☒	☐ Clinical data
☒	☐ Dual use research of concern
☒	☐ Plants

Methods

n/a	Involved in the study
☒	☐ ChIP-seq
☐	☒ Flow cytometry
☒	☐ MRI-based neuroimaging

Antibodies

Antibodies used

For immunofluorescence staining, the sections were deparaffinized, rehydrated, and blocked with 2% BSA. Primary antibodies were added and incubated overnight at 4°C. The primary antibodies used in the experiment included IL34 (MA5-17098, Thermofisher, 1:200, USA), CD31 (MA3100, Thermofisher, 1:200, USA), Cytokeratin (MA1-06312, Thermofisher, 1:200, USA), Vimentin (MA5-11883, Thermofisher, 1:250, USA), CSF1-R (PA5-115557, Thermofisher, 1:100, USA), PTP-ζ (ab126497, Abcam, 1:100, UK), Foxp3 (13-5773-82, 1:50, Thermofisher, USA), and COL1A2 (PA5-50938, 1:200, Thermofisher, USA). On the second day, the sections were washed with PBS and incubated with goat anti-mouse IgG (A10551, Thermofisher, 1:200, USA) or goat anti-rabbit IgG (A-11008, Thermofisher, 1:500, USA) secondary antibodies at room temperature for 1 hour.

Validation

Describe the validation of each primary antibody for the species and application, noting any validation statements on the manufacturer's website, relevant citations, antibody profiles in online databases, or data provided in the manuscript.

Animals and other research organisms

Policy information about studies involving animals; ARRIVE guidelines recommended for reporting animal research, and Sex and Gender in Research

Laboratory animals

Thirty-six 6-week-old C57BL/6N mice were purchased from Beijing Vital River Laboratory Animal Technology Co., Ltd. (Beijing, China). The mice were housed in standard cages under constant room temperature (23±1°C) with a 12-hour light/dark cycle and 60%-65% humidity. They had ad libitum access to food and water and were acclimatized for one week prior to the experiments. The experimental procedures and animal usage were approved by the Animal Ethics Committee of Shanghai Pudong Hospital.

Wild animals

N/A

Reporting on sex

Thirty-six 6-week-old C57BL/6N mice were purchased from Beijing Vital River Laboratory Animal Technology Co., Ltd. (Beijing, China). The mice were housed in standard cages under constant room temperature (23±1°C) with a 12-hour light/dark cycle and 60%-65% humidity. They had ad libitum access to food and water and were acclimatized for one week prior to the experiments. The experimental procedures and animal usage were approved by the Animal Ethics Committee of Shanghai Pudong Hospital.

Field-collected samples

Thirty-six 6-week-old C57BL/6N mice were purchased from Beijing Vital River Laboratory Animal Technology Co., Ltd. (Beijing, China). The mice were housed in standard cages under constant room temperature (23±1°C) with a 12-hour light/dark cycle and 60%-65% humidity. They had ad libitum access to food and water and were acclimatized for one week prior to the experiments. The experimental procedures and animal usage were approved by the Animal Ethics Committee of Shanghai Pudong Hospital.

Ethics oversight

The experimental procedures and animal usage were approved by the Animal Ethics Committee of Shanghai Pudong Hospital.

Note that full information on the approval of the study protocol must also be provided in the manuscript.

Plants

Seed stocks

N/A

Novel plant genotypes

N/A

Authentication

N/A

Flow Cytometry

Plots

Confirm that:

☒ The axis labels state the marker and fluorochrome used (e.g. CD4-FITC).

☒ The axis scales are clearly visible. Include numbers along axes only for bottom left plot of group (a 'group' is an analysis of identical markers).

☒ All plots are contour plots with outliers or pseudocolor plots.

☒ A numerical value for number of cells or percentage (with statistics) is provided.

Methodology

Sample preparation

Cell samples were washed with PBS and then resuspended for later use. Tissue samples were digested at 37°C in PBS containing 0.8 mg/mL Collagenase IV (Merck, C4-BIOC, USA) for 30 minutes. The supernatant was collected after centrifugation at 850 ×g for 10 minutes, followed by PBS washing. Dead cells were removed using Percoll (Merck, P1644, USA) according to the manufacturer's instructions, and then the samples were resuspended. The concentrations were adjusted to a concentration of 1×107 cells/mL in 100 µL of PBS for each sample. When intracellular antigens needed to be detected, cells were permeabilized for 5 minutes using 0.5% Tween 20 (Merck, P2287, USA) before incubation with the primary antibody. The samples were incubated with antibodies at 4°C and then analyzed using a flow cytometer (BD Bioscience, BD LSRFortessa, USA). Anti-Human-IL34 (MA5-17098, Thermo Fisher, USA) and Vimentin (MA5-11883, Thermo Fisher, USA) were used for screening IL34+ CAFs. FITC-Mouse-anti-Human-Granzyme B (GzmB) (20 µL/test, BD Bioscience, 560211, USA) or Alexa Fluor® 488-Mouse-anti-Human-Ki67 (5 µL/test, BD Bioscience, 561165, USA) and APC-Mouse-anti-Human-CD8 (5 µL/test, BD Bioscience, 340584, USA) were used to evaluate the cytotoxicity of CD8+ T cells. Foxp3-PE (12-5773-82, Thermo Fisher, USA) and CD8-FITC (11-0081-82, Thermo Fisher, USA) were used to detect the proportion of Tregs cells. The obtained data were analyzed using BD FACSDiva software

Instrument

Cell samples were washed with PBS and then resuspended for later use. Tissue samples were digested at 37°C in PBS containing 0.8 mg/mL Collagenase IV (Merck, C4-BIOC, USA) for 30 minutes. The supernatant was collected after centrifugation at 850 ×g for 10 minutes, followed by PBS washing. Dead cells were removed using Percoll (Merck, P1644, USA) according to the manufacturer's instructions, and then the samples were resuspended. The concentrations were adjusted to a concentration of 1×107 cells/mL in 100 µL of PBS for each sample. When intracellular antigens needed to be detected, cells were permeabilized for 5 minutes using 0.5% Tween 20 (Merck, P2287, USA) before incubation with the primary antibody. The samples were incubated with antibodies at 4°C and then analyzed using a flow cytometer (BD Bioscience, BD LSRFortessa, USA). Anti-Human-IL34 (MA5-17098, Thermo Fisher, USA) and Vimentin (MA5-11883, Thermo Fisher, USA) were used for screening IL34+ CAFs. FITC-Mouse-anti-Human-Granzyme B (GzmB) (20 µL/test, BD Bioscience, 560211, USA) or Alexa Fluor® 488-Mouse-anti-Human-Ki67 (5 µL/test, BD Bioscience, 561165, USA) and APC-Mouse-anti-Human-CD8 (5 µL/test, BD Bioscience, 340584, USA) were used to evaluate the cytotoxicity of CD8+ T cells. Foxp3-PE (12-5773-82, Thermo Fisher, USA) and CD8-FITC (11-0081-82, Thermo Fisher, USA) were used to detect the proportion of Tregs cells. The obtained data were analyzed using BD FACSDiva software

Software

Cell samples were washed with PBS and then resuspended for later use. Tissue samples were digested at 37°C in PBS containing 0.8 mg/mL Collagenase IV (Merck, C4-BIOC, USA) for 30 minutes. The supernatant was collected after centrifugation at 850 ×g for 10 minutes, followed by PBS washing. Dead cells were removed using Percoll (Merck, P1644, USA) according to the manufacturer's instructions, and then the samples were resuspended. The concentrations were adjusted to a concentration of 1×107 cells/mL in 100 µL of PBS for each sample. When intracellular antigens needed to be detected, cells were permeabilized for 5 minutes using 0.5% Tween 20 (Merck, P2287, USA) before incubation with the primary antibody. The samples were incubated with antibodies at 4°C and then analyzed using a flow cytometer (BD Bioscience, BD LSRFortessa, USA). Anti-Human-IL34 (MA5-17098, Thermo Fisher, USA) and Vimentin (MA5-11883, Thermo Fisher, USA) were used for screening IL34+ CAFs. FITC-Mouse-anti-Human-Granzyme B (GzmB) (20 µL/test, BD Bioscience, 560211, USA) or Alexa Fluor® 488-Mouse-anti-Human-Ki67 (5 µL/test, BD Bioscience, 561165, USA) and APC-Mouse-anti-Human-CD8 (5 µL/test, BD Bioscience, 340584, USA) were used to evaluate the cytotoxicity of CD8+ T cells. Foxp3-PE (12-5773-82, Thermo Fisher, USA) and CD8-FITC (11-0081-82, Thermo Fisher, USA) were used to detect the proportion of Tregs cells. The obtained data were analyzed using BD FACSDiva software

Cell population abundance

Cell samples were washed with PBS and then resuspended for later use. Tissue samples were digested at 37°C in PBS containing 0.8 mg/mL Collagenase IV (Merck, C4-BIOC, USA) for 30 minutes. The supernatant was collected after centrifugation at 850 ×g for 10 minutes, followed by PBS washing. Dead cells were removed using Percoll (Merck, P1644, USA) according to the manufacturer's instructions, and then the samples were resuspended. The concentrations were adjusted to a concentration of 1×107 cells/mL in 100 µL of PBS for each sample. When intracellular antigens needed to be detected, cells were permeabilized for 5 minutes using 0.5% Tween 20 (Merck, P2287, USA) before incubation with the primary antibody. The samples were incubated with antibodies at 4°C and then analyzed using a flow cytometer (BD Bioscience, BD LSRFortessa, USA). Anti-Human-IL34 (MA5-17098, Thermo Fisher, USA) and Vimentin (MA5-11883, Thermo Fisher, USA) were used for screening IL34+ CAFs. FITC-Mouse-anti-Human-Granzyme B (GzmB) (20 µL/test, BD Bioscience, 560211, USA) or Alexa Fluor® 488-Mouse-anti-Human-Ki67 (5 µL/test, BD Bioscience, 561165, USA) and APC-Mouse-anti-Human-CD8 (5 µL/test, BD Bioscience, 340584, USA) were used to evaluate the cytotoxicity of CD8+ T cells. Foxp3-PE (12-5773-82, Thermo Fisher, USA) and CD8-FITC (11-0081-82, Thermo Fisher, USA) were used to detect the proportion of Tregs cells. The obtained data were analyzed using BD FACSDiva software

Gating strategy

Cell samples were washed with PBS and then resuspended for later use. Tissue samples were digested at 37°C in PBS containing 0.8 mg/mL Collagenase IV (Merck, C4-BIOC, USA) for 30 minutes. The supernatant was collected after

centrifugation at 850 ×g for 10 minutes, followed by PBS washing. Dead cells were removed using Percoll (Merck, P1644, USA) according to the manufacturer's instructions, and then the samples were resuspended. The concentrations were adjusted to a concentration of 1×107 cells/mL in 100 μL of PBS for each sample. When intracellular antigens needed to be detected, cells were permeabilized for 5 minutes using 0.5% Tween 20 (Merck, P2287, USA) before incubation with the primary antibody. The samples were incubated with antibodies at 4°C and then analyzed using a flow cytometer (BD Bioscience, BD LSRFortessa, USA). Anti-Human-IL34 (MA5-17098, Thermo Fisher, USA) and Vimentin (MA5-11883, Thermo Fisher, USA) were used for screening IL34+ CAFs. FITC-Mouse-anti-Human-Granzyme B (GzmB) (20 μL/test, BD Bioscience, 560211, USA) or Alexa Fluor® 488-Mouse-anti-Human-Ki67 (5 μL/test, BD Bioscience, 561165, USA) and APC-Mouse-anti-Human-CD8 (5 μL/test, BD Bioscience, 340584, USA) were used to evaluate the cytotoxicity of CD8+ T cells. Foxp3-PE (12-5773-82, Thermo Fisher, USA) and CD8-FITC (11-0081-82, Thermo Fisher, USA) were used to detect the proportion of Tregs cells. The obtained data were analyzed using BD FACSDiva software

☒ Tick this box to confirm that a figure exemplifying the gating strategy is provided in the Supplementary Information.
